# Cas10 relieves host growth arrest to facilitate spacer retention during type III-A CRISPR-Cas immunity

**DOI:** 10.1016/j.chom.2024.11.005

**Published:** 2024-12-02

**Authors:** Naama Aviram, Amanda K. Shilton, Nia G. Lyn, Bernardo S. Reis, Amir Brivanlou, Luciano A. Marraffini

**Affiliations:** 1 Laboratory of Bacteriology, The Rockefeller University, 1230 York Ave, New York, NY 10065, USA.; 2 Laboratory of Mucosal Immunology, The Rockefeller University, 1230 York Ave, New York, NY 10065, USA.; 3 Howard Hughes Medical Institute, The Rockefeller University, 1230 York Ave, New York, NY 10065, USA.; 4 Lead contact; 5 These authors contributed equally

**Keywords:** CRISPR, bacteriophage, staphylococci, adaptive immunity

## Abstract

Cells from all kingdoms of life can enter growth arrest in unfavorable environmental conditions. Key to this process are mechanisms enabling recovery from this state. Staphylococcal type III-A CRISPR-Cas loci encode the Cas10 complex that uses a guide RNA to locate complementary viral transcripts and start an immune response. When the target sequence is expressed late in the viral lytic cycle, defense requires the activity of Csm6, a non-specific RNase that inhibits the growth of the infected cell. How Csm6 protects from infection and whether growth can be restored is not known. Here we show that growth arrest provides immunity at the population level, preventing viral replication and allowing uninfected cells to propagate. In addition, the ssDNase activity of Cas10 is required for the regrowth of a subset of the arrested cells and the recovery of the infected host, presumably ending the immune response through degradation of the viral DNA.

## eTOC

CRISPR-Cas immunity generates a memory of infection after phage infection. CRISPR-Cas10 systems use this memory to halt the growth of infected cells and prevent viral proliferation. In addition, these systems possess a nuclease activity that enables the exit from arrest, thus allowing the maintenance of the acquired memories.

## Graphical Abstract

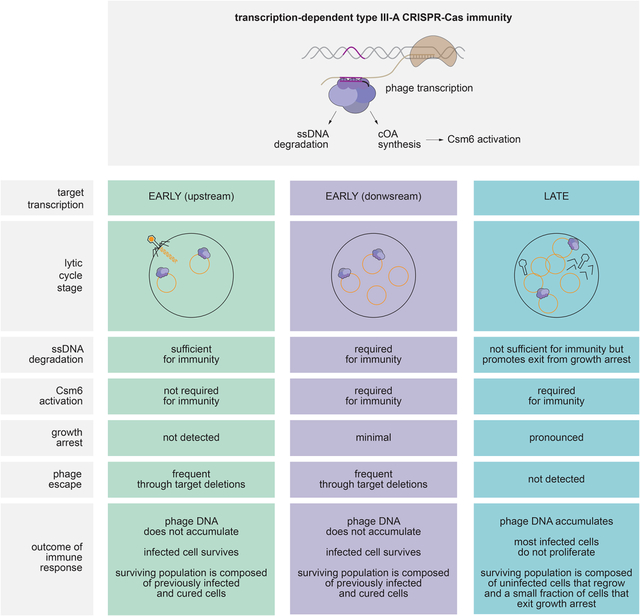

## INTRODUCTION

A fundamental adaptive tool of cells from all kingdoms of life is the ability to enter a state of growth arrest when environmental conditions are not favorable for active proliferation. Prokaryotes arrest their growth in response to environmental stress and as a defense strategy against viral (phage) infection. Immune pathways that trigger growth arrest to prevent phage propagation are collectively known as “abortive infection” mechanisms^1,2^. Whether and how infected bacteria can exit growth arrest is not known for most abortive infection responses, including those mediated by Clustered Regularly Interspaced Short Palindromic Repeat (CRISPR) loci.

CRISPR systems provide adaptive immunity to prokaryotes^3^ and consist of short repetitive sequences separated by “spacer” sequences of phage or plasmid origin^4–6^, which are acquired from the invader’s genome upon its entry into the host^3^. After being incorporated into the CRISPR locus, spacer sequences are transcribed and processed into short CRISPR RNAs (crRNAs) that are loaded on CRISPR-associated (Cas) proteins to mediate the base-pair recognition of nucleic acids. CRISPR-Cas systems are highly diverse and can be classified into seven types (I-VII) according to their *cas* gene content^7^, each of which has a different mode of immunity. Type III-A CRISPR-Cas systems rely on a multi-protein effector complex that is formed by the association of the main subunit Cas10 with four additional Cas proteins (Csm2, Csm3, Csm4, Csm5)^8–10^ and a crRNA that is used to identify complementary RNA molecules^10,11^. Target recognition activates two different domains of the Cas10-Csm complex: a nuclease domain (HD) that degrades ssDNA non-specifically^10,12,13^ and a cyclase domain (Palm) that converts ATP into cyclic tetra- or hexa-adenylates (cyclic oligoadenylates, cOA)^14,15^. These second messengers activate CRISPR-associated Rossman fold (CARF) effectors^16^. Both activities are transient, since the Csm3 subunit of the Cas10 complex cleaves the target RNA to shut off the type III response^10,11^. Type III-A CRISPR systems are commonly present in staphylococci^17,18^, where they are associated with the Csm6 CARF effector, a cOA-dependent, non-specific RNase. During phage infection of *Staphylococcus aureus*, Csm6 activation is required for immunity when the target is located on a late-expressed viral transcript^19^ and results in the degradation of both invader and host RNA molecules^20^. In contrast, crRNAs that recognize early-expressed viral transcripts do not require this RNase and presumably rely on the ssDNase activity of the Cas10-Csm complex to provide immunity. In the case of spacers that target plasmid transcripts, RNA cleavage by Csm6, as well as its ortholog Csx1 (associated with type III-B CRISPR loci in other organisms), results in growth arrest of the cell^20,21^.

It is unknown whether Csm6 activation during the type III-A CRISPR response against phage infection results in a similar growth arrest of the host and, if so, whether there are mechanisms that enable exit from this state. Perhaps more importantly, how this growth arrest affects spacer acquisition and maintenance is a central question in CRISPR-Cas immunity, as not only type III, but also type V^22,23^ and type VI^24,25^ CRISPR-Cas systems are capable of mediating immunity via growth inhibition. Hosts that acquire spacer sequences capable of mediating a halt to their own replication would be at a disadvantage to other bacteria that acquire spacers that confer protection without limiting host growth. Moreover, even if spacers that induce a growth arrest are acquired and fixed in the population, how they subsist after rounds of infection that prevent the growth of their host is unknown. Here we investigate this important question following the type III-A immune response to phage infection in staphylococci.

## RESULTS

### Spacers targeting late-expressed ΦNM4γ4 transcripts induce growth arrest of the infected cell.

**Figure 1.**
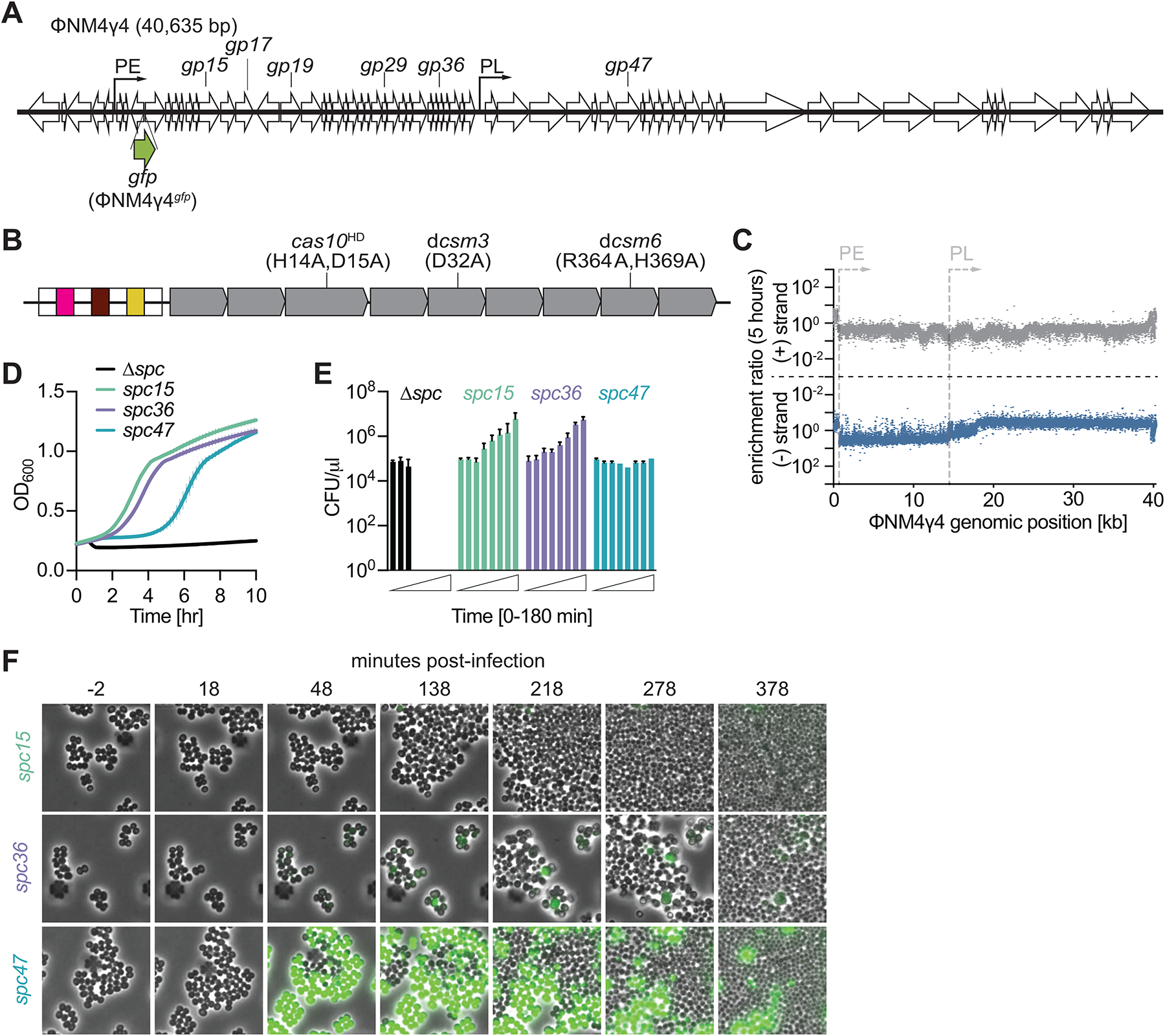
Type III-A CRISPR immunity induces the growth arrest of the infected cell. **(A)** Schematic representation of the ΦNM4γ4 phage genome. Arrows indicate promoters PE and PL. The location of the targets of the different spacers used in this study, as well as the site of insertion of *gfp* to generate ΦNM4γ4^*gfp*^, are indicated. **(B)** *S. epidermidis* type III-A CRISPR-*cas* locus. White boxes, repeats; colored boxes, spacers. Mutations introduced in this study are shown. **(C)** Enrichment ratio of spacers across the ΦNM4γ4 genome 5 hours after infection of staphylococci carrying pCRISPR. **(D)** Mean (± SD, n = 3 biological replicates) OD_600_ values of staphylococcal cultures harboring a pCRISPR plasmid programmed with different spacers after infection with ΦNM4γ4 at MOI 10. **(E)** Same as **(D)** but showing mean (± SD, n = 3 biological replicates) CFU/μl present in infected cultures, with samples taken every 30 minutes. **(F)** Fluorescence microscopy of staphylococci carrying pCRISPR plasmids programmed with different spacers infected with ΦNM4γ4^*gfp*^. See also Figure S1.

Previous work investigated the role of Csm6 in anti-viral defense using only a limited number of select spacers that targeted the staphylococcal phage ΦNM1γ6^19,26^. We decided to test the immunity mediated by a library of spacers that covered the complete sequence of the ΦNM4γ4 phage^27,28^ (Fig. 1A). We designed a plasmid library of 40,338 spacers that matched both strands of the genome every two nucleotides, and introduced it into *Staphylococcus aureus* RN4220^29^ cells harboring a second plasmid encoding the *S. epidermidis* type III-A CRISPR-Cas system without any spacers, pCRISPR (Fig. 1B). Library plasmids were isolated from cultures and spacers were amplified and analyzed by next generation sequencing (NGS) to determine their abundance. We were able to detect 40,037 unique spacer sequences, with equivalent frequency reads for most of them (Fig. S1A, the expected frequency is 1/40,338 or 2.4 × 10^−5^). We then infected the cultures with ΦNM4γ4 at a multiplicity of infection (MOI) of 2, used NGS to measure the abundance of each spacer 5 and 24 hours after phage addition, and calculated enrichment ratios as the read count post-infection divided by its abundance in the uninfected library (Figs. 1C, S1B and Table S1). As expected from the RNA targeting properties of the Cas10 complex^10,13–15,30,31^, there was little to no enrichment for spacers matching the plus strand sequences of ΦNM4γ4, which, based on the direction of the open reading frames, should not be transcribed (Fig. 1A). However, for spacers generating a crRNA complementary to predicted transcripts (i.e. sequences identical to the minus strand of the phage genome), we found that only those targeting a region spanning ~1 to 15 kb of ΦNM4γ4 were highly enriched at both timepoints post-infection, with a decrease in spacer accumulation across 15 to 18 kb (Figs. 1C and S1B). This is a surprising result given that the rest of the genome (18 to 40 kb) must be transcribed during the lytic cycle to generate RNA targets that activate the Cas10-Csm complex, and therefore should also be enriched. A previous study showed that targeting ΦNM4γ4 with a crRNA complementary to the *gp47* transcript (located at approximately 20 kb in the genome), which encodes the major head protein and is expected to be expressed late in the lytic cycle, reduced plaque formation to undetectable levels^32^. To clarify this discrepancy, we performed RNA-seq at 5, 15 and 30 minutes after ΦNM4γ4 infection (Fig. S1C and Supplementary File 1). We found that transcription of the phage genome occurs primarily from two promoters; an early promoter (PE) that directs the expression of the 1–15 kb region during the first five minutes after infection, and a second late promoter (PL) responsible for the transcription of the 15–40 kb region, starting sometime between 5 and 15 minutes after infection. These results indicate that the spacers most enriched are those that target early-expressed transcripts, and we found a strong correlation (*r*=0.59, *n*=39,796) between spacer enrichment ratios and target RNA-seq reads obtained 5 minutes post-infection (Fig. S1D).

Our previous work using select spacers against ΦNM1γ6 demonstrated that Csm6 is required for immunity mediated by spacers that target late-, but not early-expressed, viral genes^19,26^. We also found that when type III-A CRISPR immunity is triggered by a plasmid expressing a target transcript, the activation of Csm6 leads to a growth arrest of the host cell^20^. Therefore, we hypothesized that the library of spacers matching the 18–40 kb region of the phage genome trigger the activation of Csm6 and cause the growth arrest of staphylococci harboring them, preventing their enrichment. To test this, we cloned spacer sequences matching PE and PL transcripts into pCRISPR and followed the growth by optical density at 600 nm (OD_600_) of staphylococci infected with ΦNM4γ4 at MOI 10. Control cells carrying a CRISPR system with a non-targeting spacer (Δ*spc*), failed to propagate (Fig. 1D). In contrast, spacers targeting PE-derived transcripts (*spc15* and *spc36*; Fig. S1E; targeting *gp15* and *gp36*, Fig. 1A, respectively) enabled the growth of the infected cultures (Fig. 1D). The strain harboring the spacer targeting a transcript expressed from the PL promoter (*spc47*; Fig. S1E; targeting *gp47*, Fig. 1A) also survived phage infection but displayed a growth delay (Fig. 1D). To corroborate this result, we enumerated colony forming units (CFU) within the four cultures at different times after infection with ΦNM4γ4 at MOI 10 (Fig. 1E). Due to phage lysis, CFU counts drastically decreased in the absence of a targeting spacer. For cultures harboring *spc15* and *spc36*, which did not display a growth delay after infection, CFU values steadily increased. However, CFUs for the *spc47*-harboring staphylococci remained constant, at least for the first 3 hours after infection, a result that confirms the arrest, and not death, of the infected cells carrying this spacer.

Despite this cessation in proliferation, *spc47* cultures grew to high densities after infection (Fig. 1D). To investigate this in more detail, we used a virus that expresses green fluorescent protein (ΦNM4γ4^*gfp*^, Fig. 1A) and followed bacterial proliferation using fluorescence microscopy. We found that while staphylococci lacking the CRISPR-Cas system displayed green fluorescence and lysed after the addition of ΦNM4γ4^*gfp*^ (Fig. S1F), cultures harboring *spc15* continued their growth normally after infection (Fig. 1F and Supplementary video 1). When a culture harboring *spc36* was infected, some cells emitted dim fluorescence, with most staphylococci displaying a minor division stall that was followed by vigorous proliferation (Fig. 1F and Supplementary video 2). In contrast, cells producing the *spc47* crRNA turned green and stopped dividing, and uninfected cells (non-fluorescent, Fig. 1F and Supplementary video 3) took over the bacterial population around 90 minutes post-infection. We conclude that spacers targeting late-expressed transcripts mediate immunity through a growth arrest which prevents viral propagation. These spacers promote the survival of uninfected cells but impose a fitness cost for hosts that harbor them.

### Immunity triggered by early-expressed transcripts also requires Csm6 but does not induce a prolonged growth arrest

**Figure 2.**
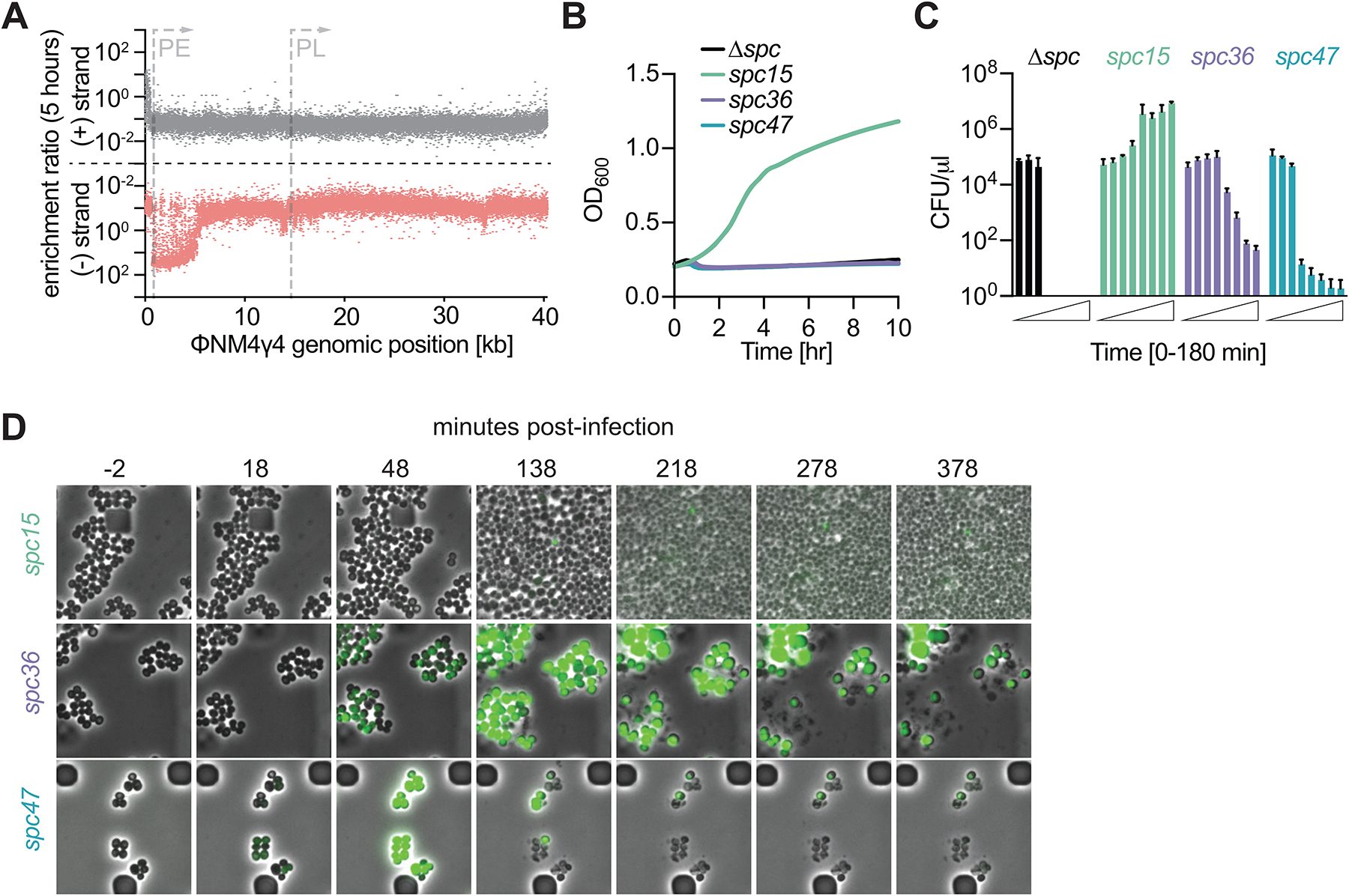
Spacers can require Csm6 for immunity without inducing a prolonged growth arrest. **(A)** Enrichment ratio of spacers across the ΦNM4γ4 genome 5 hours after infection of staphylococci carrying pCRISPR(d*csm6*). **(B)** Mean (± SD, n = 3 biological replicates) OD_600_ values of staphylococcal cultures harboring a pCRISPR(d*csm6*) plasmid programmed with different spacers after infection with ΦNM4γ4 at MOI 10. **(C)** Same as **(B)** but showing mean (± SD, n = 3 biological replicates) CFU/μl present in infected cultures, with samples taken every 30 minutes. **(D)** Fluorescence microscopy of staphylococci carrying pCRISPR(d*csm6*) plasmids programmed with different spacers infected with ΦNM4γ4^*gfp*^. See also Figure S2.

The lack of growth arrest after infection of cells that carry spacers targeting PE-derived transcripts, as well as their positive selection in our library experiments, suggest that these spacers do not activate Csm6. However, our current understanding of the molecular events that occur during type III-A CRISPR immunity indicate that, since recognition of a complementary RNA sequence results in the synthesis of cOA molecules by the Cas10 complex^14,15^, spacers targeting PE-derived transcripts should also induce the production of second messengers that activate Csm6. To study a possible role for Csm6 activation during type III-A targeting of early-expressed ΦNM4γ4 transcripts, we performed infection experiments in the absence of Csm6 activity, introducing the spacer library into staphylococci carrying a pCRISPR plasmid that expresses a catalytically dead Csm6, dCsm6^15,19,33^ (Fig. 1B). We were able to detect 39,953 spacer sequences after NGS of the resulting culture (Fig. S2A). Surprisingly, we found that approximately half of the spacers targeting early-expressed transcripts displayed lack of positive selection both at 5 and 24 hours after phage treatment (Figs. 2A and S2B). This result indicates that these spacers require the activation of Csm6 to confer immunity. Indeed, the number of unique spacer sequences derived from the minus strand that we were able to detect 24 hours after phage selection was 9,186 in the presence of dCsm6, compared to 19,859 in cultures carrying a wild-type III-A CRISPR system, with most of the missing spacers mapping to the region with low enrichment (Figs. S2C–D). This depletion suggests that many of the infected cells harboring these spacers undergo lysis in the absence of Csm6 activity. To corroborate this, we followed the growth of cultures carrying single spacers, cloned into pCRISPR(d*csm6*), and infected with ΦNM4γ4 at a MOI of 10 (Figs. 2B, S2E). We found that *spc15* mediated complete defense, equivalent to that observed in the presence of wild-type Csm6 (Fig. S2F). In contrast, staphylococci carrying *spc47* succumbed to phage infection, similarly to cells lacking a targeting spacer. Lack of defense was further corroborated both by CFU enumeration after infection (Fig. 2C) and fluorescent microscopy of ΦNM4γ4^*gfp*^-infected cells (Fig. 2D and Supplementary videos 4–5).

Consistent with the results of Figure 2A, *spc36* failed to provide immunity in the absence of Csm6 activity, as measured by optical density (Fig. 2B, S2E), CFU enumeration (Fig. 2C) and fluorescence microscopy (Fig. 2D and Supplementary video 6); the latter showing the lysis of the infected cells. Interestingly, the decrease in CFU counts was less drastic than those obtained in the presence of *spc47* and Δ*spc* pCRISPR plasmids. This result suggests that either the sequence-specific target transcript binding, target RNA cleavage, and/or non-specific ssDNA degradation by the Cas10 complex can interfere with the phage lytic cycle. We explored other targets in the region between *spc15* and *spc36*: spacers 17, 19 and 29 (Fig. S1F), which target *gp17*, *gp19* and *gp29* transcripts, respectively (Fig. 1A). We followed the growth of cultures harboring these spacers, which were infected at an MOI of 1 to increase the dynamic range of the measurement and facilitate the spread of the different growth curves. As expected, in the presence of the wild-type type III-A *cas* operon all of the new spacers provided strong immunity (Fig. S2G). In contrast, in cells expressing dCsm6 we found that the magnitude of the growth delay correlated with the target position, i.e., the closer to the early promoter, the smaller the delay (Fig. S2H). Altogether these results demonstrate that spacers targeting the downstream region of the early-expressed transcript of ΦNM4γ4 (“PE-downstream” spacers) require Csm6 activity to provide defense, with spacers targeting more downstream regions being increasingly dependent on this RNase. However, as opposed to the Csm6-dependent response of spacers targeting the late-expressed transcript (*spc47*), PE-downstream spacers do not trigger a prolonged growth arrest. This suggests the existence of a mechanism that promotes cellular proliferation after Csm6 activation, and therefore enables the enrichment of staphylococci harboring these spacers within the bacterial population.

### Cas10 ssDNase activity is required to prevent Csm6-dependent growth arrest when the target is located on an early-expressed transcript

**Figure 3.**
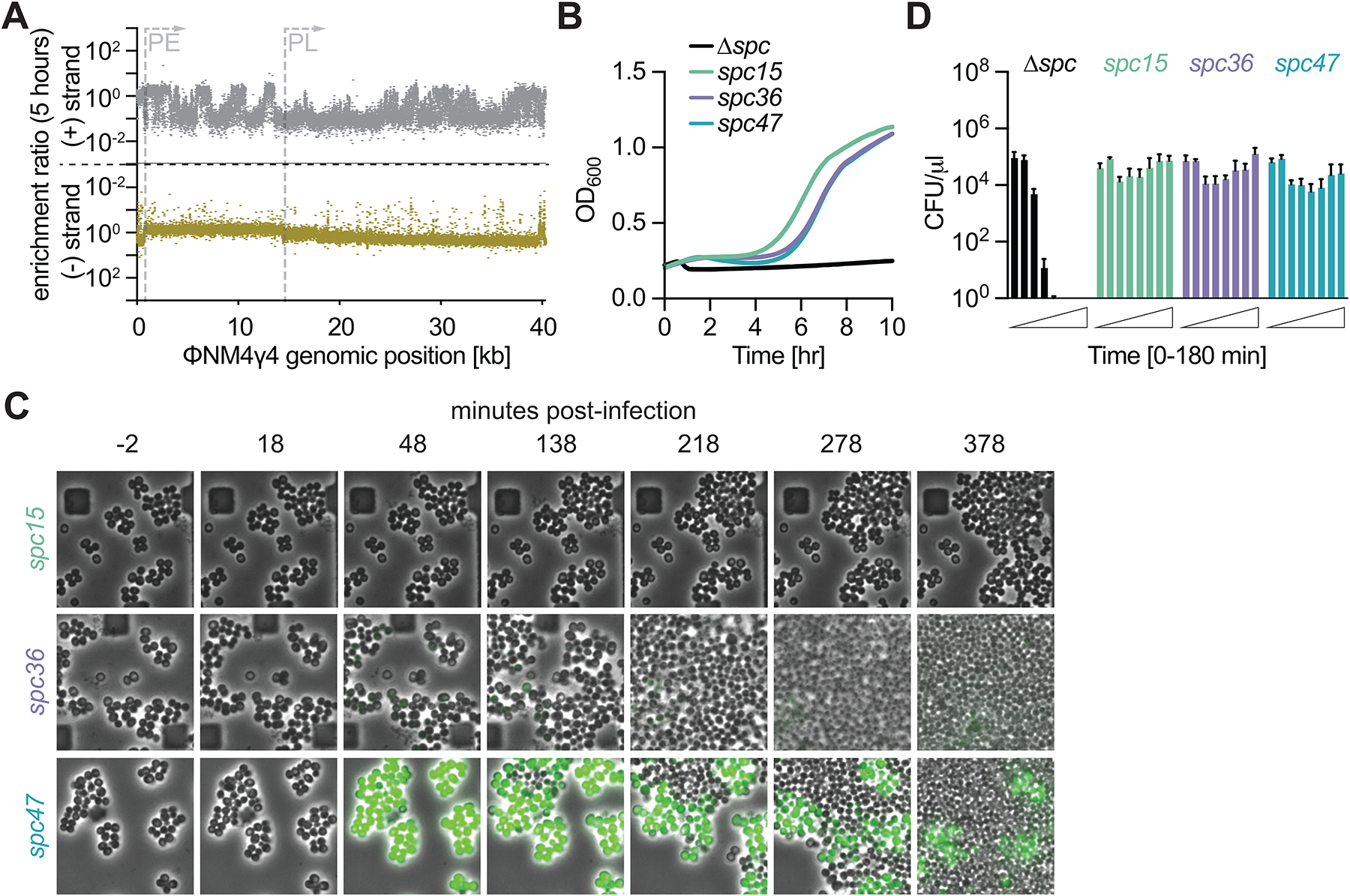
Cas10 ssDNase activity is required for regrowth after infection. **(A)** Enrichment ratio of spacers across the ΦNM4γ4 genome 5 hours after infection of staphylococci carrying pCRISPR(*cas10*^HD^). **(B)** Mean (± SD, n = 3 biological replicates) OD_600_ values of staphylococcal cultures harboring a pCRISPR(*cas10*^HD^) plasmid programmed with different spacers after infection with ΦNM4γ4 at MOI 10. **(C)** Fluorescence microscopy of staphylococci carrying pCRISPR(*cas10*^HD^) plasmids programmed with different spacers infected with ΦNM4γ4^*gfp*^. **(D)** Same as **(B)** but showing mean (± SD, n = 3 biological replicates) CFU/μl present in infected cultures, with samples taken every 30 minutes. See also Figure S3.

The absence of GFP fluorescence in most *spc15*- and *spc36*-harboring staphylococci after infection suggests that the CRISPR response within these cells can prevent the transcription of the ΦNM4γ4 genome, even of sequences expressed early in the lytic cycle. Previously we showed that the nuclease activity of Cas10 is required to eliminate target plasmid DNA during *S. epidermidis* type III-A CRISPR-Cas immunity^20^. Therefore, we hypothesized that this activity could be important for inactivating the viral DNA as well. To test this, we performed infection experiments in staphylococci carrying a mutation in *cas10* that abolishes nuclease activity, Cas10^HD10,20^ (Fig. 1B). We introduced the spacer library into these cells and were able to detect 40,079 spacer sequences after evaluation of the resulting culture by NGS (Fig. S3A). We found that targeting spacers were not enriched, neither at 5 (Fig. 3A) nor at 24 hours (Fig. S3B) after phage treatment. Interestingly, there was a small but significant enrichment observed for spacers targeting PL-derived transcripts (Fig. 3A; average enrichment of spacers targeting PE-transcripts, 0.78±0.26; of PL-derived transcripts, 1.96±0.73; *p*<0.0001, Welch’s *t*-test). We hypothesize that this is a consequence of the rapid activation of Csm6 by crRNAs complementary to PE-transcripts, which leads to an earlier growth arrest (and therefore less cell proliferation) than those that target PL-derived phage RNAs.

Given that Csm6 should be activated in the absence of Cas10 nuclease activity, we hypothesized that spacers should be able to provide the population-level immunity mediated by this RNase. To test this, we performed phage infection experiments of cultures harboring a single spacer and the *cas10*^HD^ allele at MOI 10. In the presence of *spc15*, *spc36* or *spc47*, addition of ΦNM4γ4 resulted in a delay in the increase of OD600 (Fig. 3B), remarkably similar to the growth curve of cultures harboring *spc47* in a wild-type *cas10* background (Fig. S3C), for which immunity depends on Csm6. Indeed, the same experiment in the presence of *cas10*^HD^/d*csm6* double mutant showed the complete absence of defense, with cultures collapsing upon phage addition (Fig. S3D).

Fluorescence microscopy of *cas10*^HD^ cells infected with ΦNM4γ4^*gfp*^ showed that staphylococci harboring *spc15* did not express GFP, yet stalled their proliferation after infection (Fig. 3C and Supplementary video 7). Similarly, cells equipped with *spc36* showed very dim and non-uniform green fluorescence and stopped growing after phage infection (Fig. 3C and Supplementary video 8). We hypothesize that the lack of green fluorescence signal is a consequence of early Csm6-mediated degradation of the *gfp* phage transcript. In contrast, staphylococci that harbored *spc47* in the absence of Cas10 nuclease activity showed strong green fluorescence during their growth arrest (Fig. 3C and Supplementary video 9), similarly to the cells that carry wild-type *cas10* (Fig. 1F and Supplementary video 3), with uninfected cells that do not express GFP taking over the bacterial population. Finally, these results were corroborated by enumerating CFUs at different time-points after infection at MOI 10, an experiment that showed a constant number of cells over time regardless of the spacer sequence (Fig. 3D). Altogether these experiments confirm that the immunity mediated by *spc47* has identical properties in the presence or absence of the Cas10 nuclease activity, demonstrating that this immunity is mediated exclusively by Csm6. In contrast, the Cas10 nuclease activity is fundamental for the immunity provided by *spc15* and *spc36*, and promotes the regrowth of the infected cells. When this activity is eliminated, these spacers rely on Csm6 and convey defense at the population level.

### The type III-A CRISPR-Cas response is similar across related staphylococcal phages.

To expand the results obtained for ΦNM4γ4 infections, we generated a library of 40,066 spacers that matched both strands of the genome, every two nucleotides of a related staphylococcal phage, ΦNM1γ6^26^. Multiple alignment^34^ between ΦNM1γ6 and ΦNM4γ4 resulted in 77% overall homology, with 54% of their genomes being over 95% identical^35^. Both phages also share a similar genomic structure, containing two promoters for the transcription of early and late genes during the lytic cycle (Fig. S4A), an expression pattern that we corroborated by performing RNAseq at different times after infection of staphylococci with ΦNM1γ6, in the absence of immunity (Fig. S4B). We first infected cells harboring the spacer library and a wild-type CRISPR-Cas locus at MOI 2 and calculated the enrichment ratios as before. We found a very similar pattern to that obtained for the library targeting ΦNM4γ4, with the most efficient spacers located across the region transcribed from the early promoter, both at 5 (Fig. S4C) and 24 (Fig. S4D) hours after infection. We also measured spacer enrichment at 5 (Fig. S4E) and 24 (Fig. S4F) hours after infection of strains carrying the d*csm6* mutation, and found similar enrichment distributions to those obtained when targeting ΦNM4γ4, with a set of “PE-downstream” spacers that require Csm6 activity to provide defense. Likewise, infection of staphylococci expressing the nuclease dead Cas10^HD^ at 5 (Fig. S4G) and 24 (Fig. S4H) resulted in a slight depletion of spacers targeting the early-expressed transcript.

Finally, we tested the role of Csm3 in the type III-A CRISPR-Cas response during library infections with ΦNM1γ6 by introducing a mutation (D32A, which generates a “dead” Csm3, dCsm3; Fig. 1B) that prevents target RNA cleavage^11,13,19^. Given that Csm3 activity leads to the deactivation of the Cas10 complex and stops the production of cOAs, this RNase could contribute to stop, and possibly revert, the growth arrest mediated by Csm6. If so, infections performed in a d*csm3* genetic background could prolong the growth arrest and affect spacer enrichment patterns. We engineered CRISPR-*cas* loci containing d*csm6*/d*csm3* (Figs. S4I–J) or *cas10*^HD^/d*csm3* (Figs. S4K–L) mutations and treated the cultures with ΦNM1γ6 at MOI 2 for 5 (Figs. S4I, K) or 24 (Figs. S4J, L) hours. We did not find changes in the distribution of spacer enrichment across the viral genome, a result that indicates a minimal role for Csm3 target cleavage in the outcome of the type III-A CRISPR-Cas response.

### Spacer acquisition during the type III-A CRISPR response is biased against sequences that mediate growth arrest

**Figure 4.**
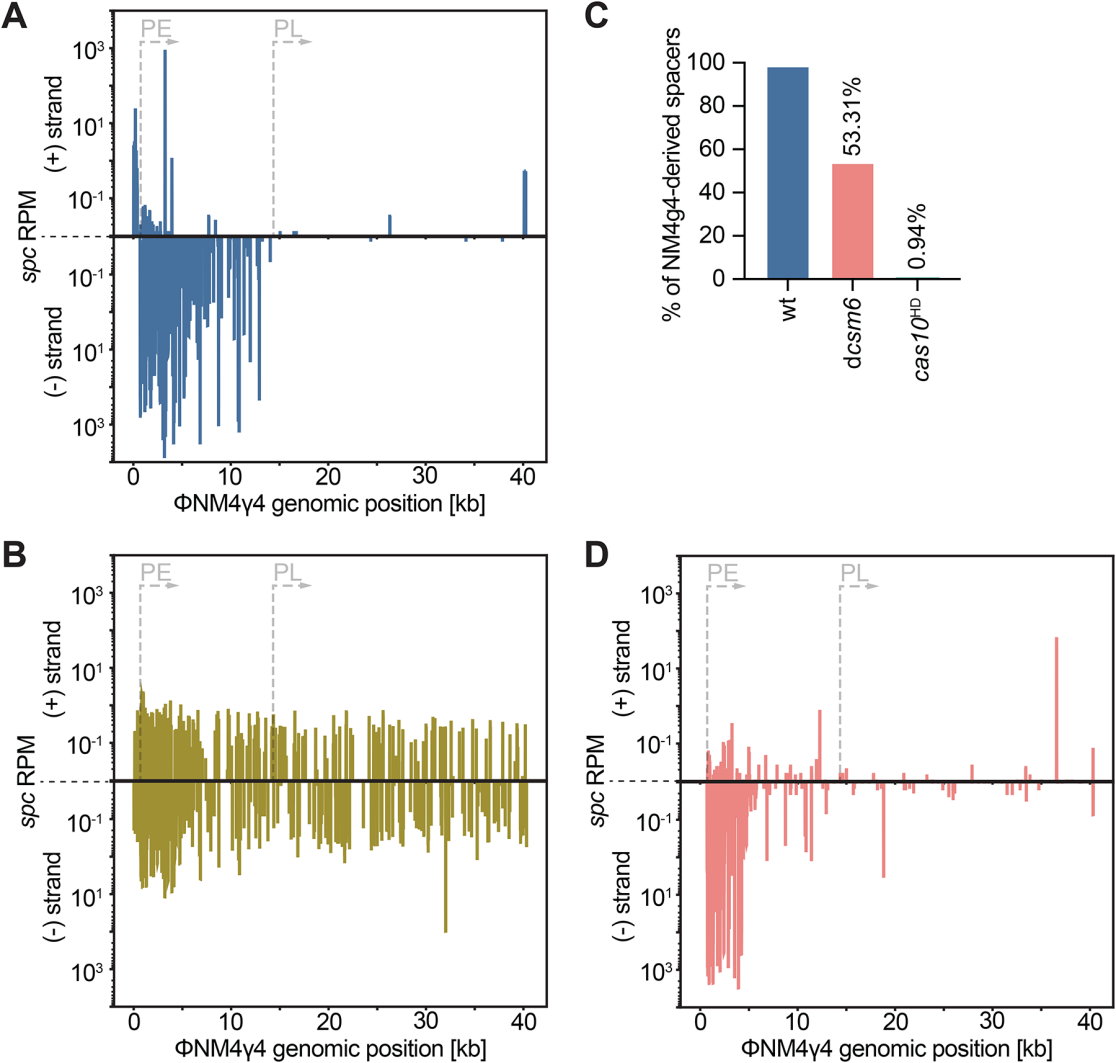
Spacers that mediate growth arrest are rarely acquired. **(A)** Reads per million (RPM) of spacer sequences acquired after infection of staphylococci harboring pCRISPR with ΦNM4γ4, plotted by genomic position. **(B)** Same as **(A)** but following infection of staphylococci harboring pCRISPR(*cas10*^HD^). **(C)** Percent of acquired spacers derived from ΦNM4γ4. **(D)** Same as **(A)** but following infection of staphylococci harboring pCRISPR(d*csm6*).

The targeting properties of different spacers should affect their abundance and retention during the type III-A CRISPR-Cas response against phages, in principle leading to a robust selection of acquired PE-targeting spacers that enable host survival but a very low frequency of acquisition for PL-targeting spacers that trigger growth arrest. To test this, we infected cells harboring a pCRISPR plasmid with a single repeat and no spacers^36^ with ΦNM4γ4 at an MOI of 2 in semi-solid agar plates (conditions that were previously shown to minimize the rise of non-CRISPR, phage-resistant colonies^37^). The sequences of the newly acquired spacers within surviving colonies were obtained via NGS and mapped to the phage genome. The majority of spacers were inserted in the orientation that, when the CRISPR array is transcribed, would generate a crRNA complementary to PE-generated phage RNA (Fig. 4A, “minus strand” spacers, and Supplementary File 1). We also found some spacer acquisition from the plus strand, which could provide early immunity given the transcription of RNA targets from this region during the first 15 minutes of infection (~0–5 kb, Fig. S1C). Therefore, these results suggest that acquired spacer sequences are selected and retained for their ability to provide immediate defense. Alternatively, there could be a promoter-specific effect on spacer acquisition, where sequences from the DNA transcribed from PE, but not those from the DNA transcribed by PL, are uniquely captured by the Cas1-2 integrase complex^38,39^. Transcription-dependent spacer acquisition, which leads to the preferential incorporation of spacers from rDNA and tDNA genes, was found in both *S. aureus* and *Streptococcus thermophilus* that harbor type III-A systems^36,40^. To explore these different possibilities, we performed spacer acquisition experiments using pCRISPR carrying *cas10*^HD^, a genetic background in which spacers that produce crRNAs targeting both PE- and PL-derived viral transcripts mediate growth arrest and population-level immunity (Figs. 3B–D). We found that spacer acquisition occurred at a very low frequency from both the minus and plus strand (Fig. 4B and Supplementary Table 1), with the majority of the acquired spacers originating from the pCRISPR(*cas10*^HD^) plasmid and only 0.94% matching the phage genome (in the wild-type genetic background 97.96% were derived from the virus; Fig. 4C). These data demonstrate that there is a severe bias against the incorporation of spacers that mediate growth arrest, regardless of how and/or when their targets are transcribed. Finally, to corroborate the importance of Cas10-mediated immunity in spacer acquisition, we performed experiments in the d*csm6* genetic background, where only spacers matching the upstream region of the PE operon provide strong defense in our library analysis (Fig. 2A). The distribution of acquired spacers was similar to that of the enrichment within the spacer library after infection (minus strand, Fig. 4D, and Supplementary Table 1). Altogether these results demonstrate that spacer acquisition by type III-A CRISPR systems displays a strong preference for sequences that mediate immunity through Cas10 ssDNase activity and enable the proliferation of the infected cells.

### Cas10 ssDNase activity is required to relieve the growth arrest of infected cells

Interestingly, thirty-nine spacers were acquired from the PL-transcribed region of the phage (Supplementary Table 1) during the wild-type type III-A CRISPR-Cas response. In addition, previous bioinformatic analyses that described type III-A CRISPR loci in environmental and clinical staphylococcal isolates^17,18^ reported the presence of spacers that are predicted to target late-expressed phage genes (Table 1). To maintain spacers that induce growth arrest within the population after their acquisition, non-proliferating cells should be able to resume growth sometime after infection. A requisite for this would be the clearance of the viral genome to stop the transcription of the target RNA, which in turn stops Csm6 activation due to the following: (i) there would be no new target transcripts that will induce the production of cyclic oligoadenylates; (ii) old target transcripts are cleaved by Csm3 and cannot induce the production of cyclic oligoadenylates; and (iii) Csm6 has ring nuclease activity that degrades its ligand immediately after binding^41^. Previous work indicated that Cas10 ssDNase activity is responsible for the clearance of plasmid DNA during the type III-A response^20^ and therefore could be responsible for phage DNA degradation. To test this, we performed qPCR to measure the relative amounts of viral DNA in cultures expressing either wild-type Cas10 or Cas10^HD^. In the absence of immunity (no spacer control, Δ*spc*) cultures displayed a rapid increase in phage DNA levels for the first 3 hours post infection, after which the culture lysed and it was not possible to obtain cells for DNA extraction (Fig. S5A). In the presence of type III-A immunity phage DNA levels were much lower.

**Figure 5.**
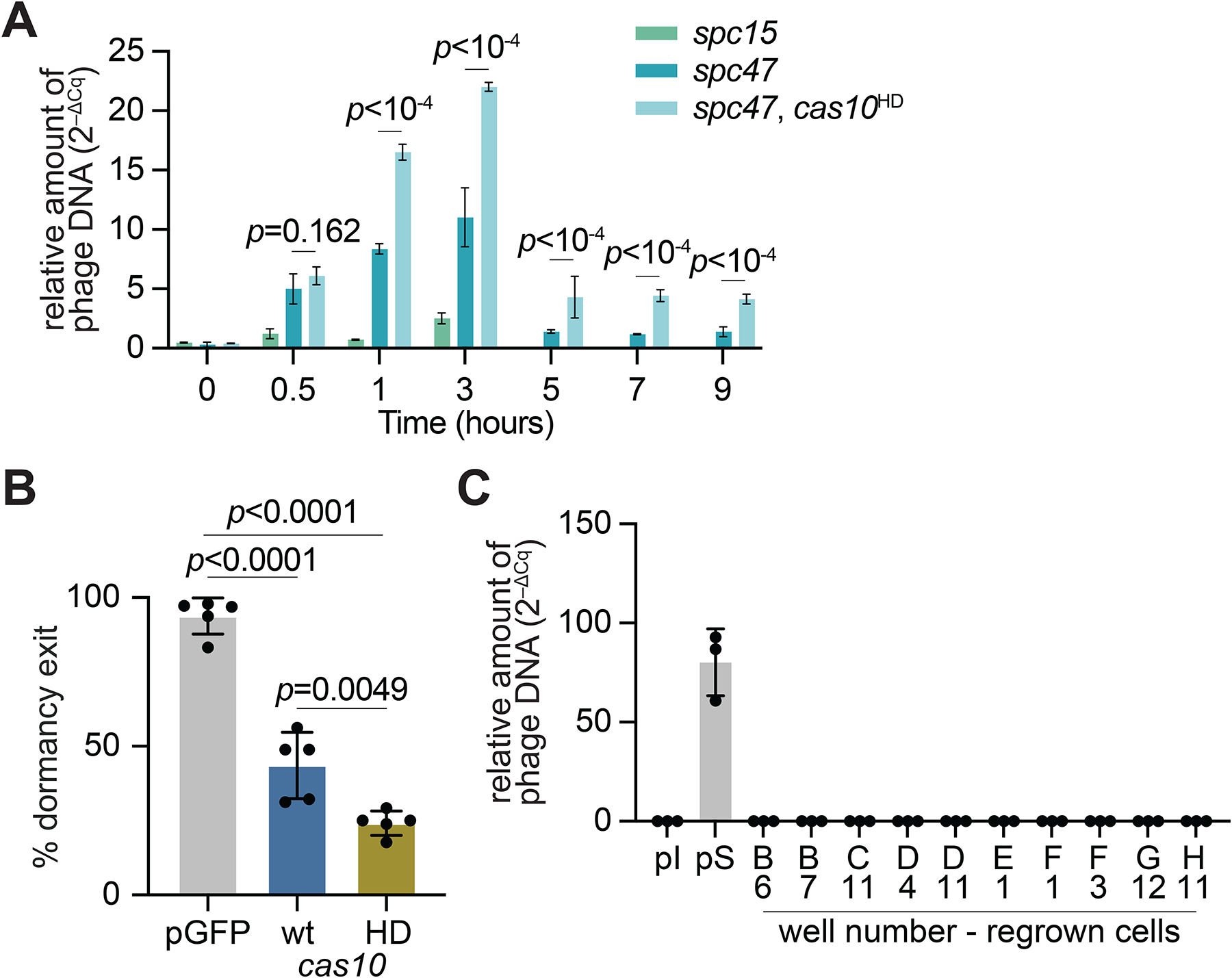
Cas10 ssDNase activity is required to relieve the growth arrest of infected cells. **(A)** Mean (± SD, n = 3 technical replicates) relative abundance of phage DNA, measured by qPCR, after infection of staphylococci harboring pCRISPR or pCRISPR(*cas10*^HD^) programmed with different spacers, with ΦNM4γ4^*gfp*^ at MOI 10. *p* values were calculated with Two-Way ANOVA and Tukey’s multiple comparisons tests. **(B)** Mean (± SD, n=5 independent experiments) percentage of single cells infected with ΦNM4γ4^*gfp*^ that grew after index-sorting into a 96-well plate. Uninfected staphylococci expressing GFP (pGFP) were used as control. *p* values were calculated with One-Way ANOVA and Tukey’s multiple comparisons tests. **(C)** Same as **(A)** but using DNA extracted from cultures obtained in **(B)**. “pI”, pre-infection culture; “pS”, pre-sorting infected culture; the other samples are re-grown cultures taken from the indicated plate well. See also Figure S5.

Compared to *spc15*, *spc47* cultures showed an increase in qPCR values during the first three hours of infection which was followed by a drastic decrease (Fig. 5A). This is most likely due to two factors: (i) the growth of uninfected cells and consequent dilution of arrested cells containing phage genomes, and (ii) the clearance of phage genomes by Cas10 over time. The contribution of the nuclease was corroborated by the qPCR values obtained from *spc47*/*cas10*^HD^ cultures, which were significantly higher than those from infected bacteria expressing wild-type Cas10 (Fig. 5A). These results demonstrate the importance of the ssDNase of Cas10 for the clearance of phage DNA, both early and late in the infection cycle.

Next we investigated whether phage DNA clearance could enable infected cells harboring *spc47* to exit from growth arrest. We followed individual cells emitting green fluorescence via microscopy (Supplementary video 3) to determine if they divide after infection with ΦNM4γ4^*gfp*^. However, the vigorous regrowth of uninfected cells saturated the field of view and prevented us from distinguishing the fate of individual infected cells (Fig. 1F). To circumvent this issue, we performed fluorescently activated cell sorting (FACS) of infected *spc47* cultures to isolate single staphylococci undergoing cell arrest and monitor their growth. We first sorted cells into 96-well plates containing rich media. The sort was gated by the GFP signal of the cells (Fig. S5B), enabling us to remove the uninfected (i.e. non-fluorescent) cells. Uninfected cultures of mixed cells carrying a GFP- or mCherry-expressing plasmid were used as a sorting control (Fig. S5C–D). Following incubation of the plates at 37°C and measurement of OD_600_ values, we detected growth in ~44% of the wells (Fig. 5B). Compared to the growth of uninfected sorted cells carrying the GFP-expressing plasmid (Fig. S5E), the re-growth of the sorted infected cells (Fig. S5F) was more variable, starting approximately two hours later than the uninfected control (Fig. S5G). Viral DNA was undetectable by qPCR in the grown cultures (Fig. 5C), a result that suggests the curing of phage infection. We wondered whether the exit from growth arrest was inversely correlated with the burden of the ΦNM4γ4^*gfp*^ lytic cycle for the host cell. We compared the average of the initial green fluorescence value for staphylococci that did or did not regrow in the 96-well plate after cell sorting, and found a significant increase in GFP expression levels in cells that were unable to proliferate (Fig. S5H). In contrast, the time each arrested cell took to resume growth was not correlated to initial GFP intensity (Fig. S5I). We also monitored individual sorted cells under the microscope and confirmed that some staphylococci eventually lose GFP signal and begin division (Fig. S5J). We hypothesized that the nuclease activity of Cas10 could enable this escape from the growth arrest. We therefore repeated FACS with infected staphylococci that harbored *spc47* and *cas10*^HD^ and detected bacterial growth in less than 25% of wells (Fig. 5B). Interestingly, in the absence of Cas10 nuclease activity, the initial GFP intensity no longer correlated to the chances of regrowth (Fig. S5K). Overall, these data demonstrate that the nuclease activity of Cas10 contributes to the retention of spacers that target late-expressed phage transcripts.

### Csm6-induced growth arrest provides broad-spectrum immunity.

**Figure 6.**
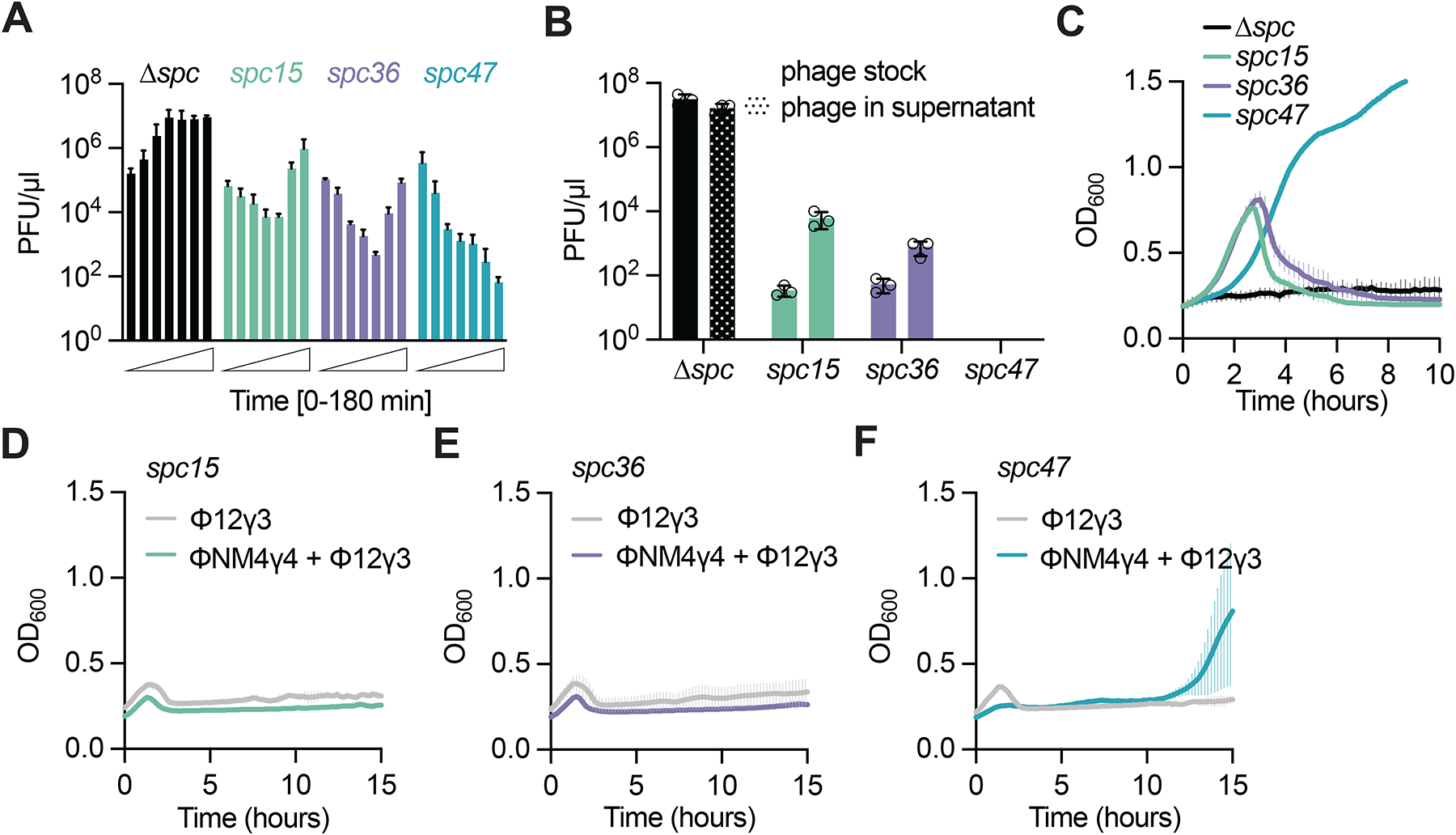
Csm6-induced growth arrest provides broad-spectrum immunity. **(A)** Mean (± SD, n = 3 biological replicates) PFU/μl in the supernatant of cultures harboring pCRISPR programmed with different spacers, sampled every 30 minutes after infection with ΦNM4γ4 at an MOI of 10. **(B)** Mean (± SD, n = 3 biological replicates) PFU/μl in supernatants of cultures shown in **(A)** (taken at the 3-hour time point) or from a phage stock spotted on top agar containing staphylococci harboring pCRISPR plasmids programmed with different spacer sequences. **(C)** Mean (± SD, n = 3 biological replicates) OD600 values of staphylococcal cultures after infection with ΦNM4γ4 at MOI 10 for one hour in panel **(B)**, re-diluted to OD_600_ of 0.1. **(D-F)** Mean (± SD, n = 3 biological replicates) OD_600_ values of staphylococcal cultures harboring pCRISPR programmed with *spc15* **(D)**, *spc36* **(E)** or *spc47* **(F)**, infected with ΦNM4γ4 at MOI 10 for one hour and then infected with Φ12γ3 at MOI 10. Infection with Φ12γ3 without prior treatment is shown as control. See also Figure S6.

The occurrence of spacers that produce crRNAs complementary to late-expressed targets in *Staphylococcus* isolates^17,18^ suggests that these spacers confer evolutionary advantages that offset the fitness cost imposed by the growth arrest triggered by these sequences during the type III-A defense. Since we previously showed that Csm6 was important to maintain immunity against phages containing mutations in their target sequence^19^, we hypothesized that the population immunity mediated by this RNase could protect against the rise of mutant phages with nucleotide substitutions that prevent the complete annealing of the crRNA to its target transcript and enable evasion of type III-A CRISPR immunity. To investigate this, we measured ΦNM4γ4 proliferation through the enumeration of plaque-forming units (PFU) at different times after infection of cultures carrying *spc15, spc36* and *spc47* at an MOI of 10. A non-targeting control culture (Δ*spc*) displayed a continuous PFU increase. In contrast, cultures carrying *spc15* and *spc36* showed an initial decrease in the production of phage particles followed by a surge in PFU approximately 2.5 hours after infection (Fig. 6A). To test for the presence of phage escapers in these cultures, we amplified and sequenced the *spc15* and *spc36* targets using DNA extracted from 10 individual plaques. We found that all these phages contained deletions that spanned the target sequence (Fig. S6A). We quantified these escapers and found that the great majority of phages in the supernatants can escape type III-A defense (Fig. 6B). Surprisingly, despite the presence of *spc15* and *spc36* escaper phages in infected cultures, the overall growth and viability of the bacterial population was unaffected (Fig. 1D). We hypothesized that the escaper phages reach high numbers during stationary growth when cells are not hospitable to infection^42^. To test this possibility, we diluted previously infected *spc15* and *spc36* cultures that had reached stationary phase to an OD of 0.1. The diluted cells resumed exponential growth and became susceptible to infection by phage escapers, which bypassed type III-A CRISPR immunity and caused the collapse of the population (Fig. 6C).

In contrast to the detrimental rise of ΦNM4γ4 escapers in cultures harboring *spc15* or *spc36*, no escaper plaques were detected in supernatants of *spc47* cultures, in agreement with the steady decrease of PFU over time, (Fig. 6B). In addition, the growth of infected *spc47* cultures that were diluted to OD 0.1 was not affected (Fig. 6C). These results suggest that escaper phages cannot propagate in bacterial populations that, due to the prior activation of Csm6, contain a large fraction of arrested cells. If this is correct, *spc47*-mediated type III-A CRISPR immunity should also protect the host against other unrelated phages that do not have a target sequence for *spc47* crRNA. To test this, we performed sequential infection of staphylococci harboring *spc15*, *spc36* or *spc47* with ΦNM4γ4 at MOI 10 and subsequently Φ12γ3^43^, which lacks target sequences for these spacers. While *spc15* and *spc36* cultures succumbed to Φ12γ3 treatment (Figs. 6D–E), staphylococci carrying *spc47* were able to survive the double infection (2/3 replicates; Figs. 6G and S6B). As expected, this cross-immunity was not observed in staphylococci expressing dCsm6 (Fig. S6C). Similar results were obtained using Φ80α*vir*^35^ as the non-targeted phage (Figs. S6D–F). Together, these results demonstrate that the Csm6-induced growth arrest generated provides broad spectrum immunity against other phages present in the host environment.

## DISCUSSION

Here we thoroughly characterized the type III-A CRISPR-Cas immune response to phage infection in staphylococci. On one hand, when the crRNA recognizes the invading phage early after infection, immunity relies on ssDNA degradation by the Cas10-Csm complex, which is believed to attack substrates present at transcription bubbles or in viral replication intermediates to prevent the progression of the lytic cycle^12,13^. This is sufficient to defend the individual cell which is rapidly “cured” from infection. On the other hand, Csm6 is required for defense when the target transcript is expressed late in the phage lytic cycle. It generates a prolonged growth arrest of the infected cells, that prevents phage propagation and allows the survival of uninfected cells. By isolating individual infected cells in the arrested state, we found that about half of them can resume growth many hours after being exposed to phage. We determined that regrowth is in part enabled by the ssDNase activity of the Cas10-Csm complex. We found that arrested cells begin to replicate again approximately two hours after an uninfected control (Fig. S5H). In contrast, uninfected cells can proliferate throughout the course of infection, and therefore the majority of bacteria that make up the surviving population most likely have avoided infection. Even if it enables the recovery of a very small number of cells, exit from growth arrest would be crucial to retain the spacers harbored by these bacteria. Given that only half of the arrested cells can resume growth, successive rounds of infection would further reduce the number of cells that were able to exit arrest after viral attack. Therefore, we hypothesize that subsequent evolutionary events, related or unrelated to phage infection, would be required to modify the genetic composition of the bacterial community and expand the number of cells carrying growth arrest-inducing spacers to fix them in the population. Once these spacers are present in a sizeable fraction of the community, they could be maintained due to the selection advantages we found they can provide, namely broad-spectrum immunity against not only the targeted virus, but also escaper phages with target mutations and unrelated phages. We also investigated the role of target RNA cleavage by Csm3 in type III-A immunity, in our spacer library experiments, and found its activity has minimal importance for defense. This could be a consequence of the presence of many copies of the target transcript (of which only a fraction are cleaved), and many copies of the phage genome that continue to transcribe new targets. If this is correct, destruction of a sparse number of target transcripts by Csm3 would have a limited effect on cOA production and in the maintenance and/or exit from the arrested state. Finally, we measured spacer acquisition into the type III-A CRISPR locus during phage infection. We found that the fixation of newly incorporated spacers that trigger Csm6-mediated prolonged growth arrest occurs at extremely low frequencies in the surviving population, a result consistent with the long time it takes for the infected cells carrying these spacers to resume growth. Since this regrowth depends partly on Cas10’s ssDNase activity, we propose that type III CRISPR-Cas systems possess a built-in mechanism to allow the subsistence of these spacers after they are acquired. In contrast, spacers that produce crRNAs that recognize early-expressed transcripts and either do not depend on Csm6 for immunity, or require Csm6 but the growth arrest is rapidly mitigated, are persistently acquired and retained. Given that our experimental system relies on the over-expression of the type III-A CRISPR-*cas* locus via a medium copy number plasmid^44^, some of the quantitative aspects of the results described above, such as the length of the arrested state and the fraction of cells that can exit from it, could be different in a native scenario where cells harbor a single chromosomal copy of the locus.

An unexpected discovery of our work is the existence of a set of spacers, such as *spc36*, that target the downstream region of the early-expressed ΦNM4γ4 transcript and require the nuclease activities of both Cas10 and Csm6 for immunity. Csm6-dependent spacers were previously thought to lead exclusively to population immunity, however our data reveals that, depending on the context, Csm6 activation can also support individual immunity to the infected cells if it is complemented by the Cas10 ssDNase to alleviate the growth arrest it causes. One possible explanation for this observation is that the downstream region of the early-expressed ΦNM4γ4 transcript showed lower abundance of reads in our RNA-seq experiments at 5 minutes post-infection (Fig. S1C). This could decrease the efficiency of the ssDNase activity in the *spc36* region (15 to 18 kb of the ΦNM4γ4 genome, Fig. 1C), as it was shown for plasmid DNA degradation by the staphylococcal type III-A CRISPR-Cas system when the target is poorly transcribed^20^.

Type III CRISPR-Cas systems are extremely complex and as a result they provide equally intricate modes of defense. We believe that the different type III immune mechanisms elicited by early- and late-transcribed targets are applicable to other phages. Indeed, this is the case for the *Sulfolobus* type III-B response against the SIRV2 and SIRV3 archaeal viruses^45,46^. Although transcription of a phage genome may not be so precisely defined by two promoters, as is the case for ΦNM4γ4 or ΦNM1γ6, most viruses have differential expression patterns to ensure the sequential completion of the different stages of the lytic cycle^47–51^. This is a problem for type III CRISPR immunity since the appearance of a target RNA late in infection forces a delayed response which is mounted when phage replication is well under way and the host cell is possibly compromised. Evolutionary evidence of this problem is found in the ubiquitous presence of many different CARF effectors associated with type III systems^52^. To date, all of the CARF effectors that have been characterized experimentally are capable of causing cell toxicity when activated by cOAs^20,53–57^, suggesting that these mediate a mechanism of immunity which saves the population but not the compromised infected host. There are also other type III CRISPR-Cas systems that may behave differently than those present in staphylococci. For example, only 36% of type III systems (70% of type III-A) encode Cas10 variants that either lack an HD domain^58^ or do not display detectable ssDNase activity^59^. In such instances, exit from growth arrest would rely exclusively on host nucleases or other processes that lead to the elimination of the viral genome from the arrested cell, as is the case for the type VI-A CRISPR-Cas system of *Listeria* which requires the activity of restriction systems to alleviate host arrest caused by Cas13^60^. In addition, many type III CRISPR loci harbor ring nucleases that degrade cOA ligands to limit the activation of CARF effectors^61,62^. How these ring nucleases modulate the type III response to affect the exit from growth arrest is not known. Finally, the presence of multiple type III CRISPR loci is common among bacterial and archaeal genomes^63^, adding another layer of complexity to the type III response which remains to be understood. For example, the hyperthermophilic bacterium *T. thermophilus* HB27c harbors, in addition to types I-B and I-C CRISPR-Cas systems, type III-A and III-B loci with three different CARF effector genes^64^. In this bacterium, spacers provide immunity only against targets located in an early-expressed region of the phage phiFa, but against targets transcribed across the complete genome of the phiKo phage; in both cases without producing a detectable growth arrest or host toxicity^65^. A recent study of the *Streptococcus thermophilus* type III-A system, which coexists with two type II and a type I CRISPR locus, and contains two copies of *csm6*, also failed to detect a growth delay in infected cultures^66^. Finally, given that previous studies have found synergistic effects between CRISPR and other immune pathways^67–69^, it is possible that the type III-A CRISPR response that we characterized in the absence of other defenses, could have different outcomes in hosts that harbor genomic defense islands.

## RESOURCE AVAILABILITY

### Lead contact

Requests for further information and resources should be directed to and will be fulfilled by the lead contact, Luciano Marraffini (marraffini@rockefeller.edu).

### Materials Availability

All unique phages, bacteria, and plasmids generated in this study are available from the lead contact without restrictions.

### Data and Code Availability

Deep sequencing and RNA-seq data are deposited in NCBI Sequence Read Archive (SRA) PRJNA1075789 and PRJNA1173170 for experiments done with ΦNM4γ4 and ΦNM1γ6, respectively. Microscopy videos used for making Figures S1F and S5J, code and any additional information required to reanalyze the data reported in this paper is available upon request from the lead contact without restrictions.

## STAR METHODS

### EXPERIMENTAL MODEL AND STUDY PARTICIPANT DETAILS

#### Bacterial strains and growth conditions

*S. aureus* RN4220^29^ cells were incubated in brain-heart-infusion (BHI) broth at 37°C shaking (220 RPM). Cultures were supplemented with chloramphenicol (10 μg/mL) to maintain CRISPR plasmids, and with spectinomycin (250 μg/mL) to maintain the spacer-library plasmids. *E. coli* Endura cells (Biosearch Technologies; Cat# 60242) were incubated in Lysogeny broth (LB) at 37°C shaking (220 RPM). Cultures were supplemented with spectinomycin (100 μg/mL) to maintain the spacer-library plasmids. All bacteriophages used in this study are listed in Supplementary Table S1 under “Bacterial strains and phages”.

### METHOD DETAILS

#### Molecular cloning

All plasmids and oligonucleotides used in this study are listed in Supplementary Table S1. Detailed cloning strategies are described in Supplementary Table S1 under “Cloning strategies”.

#### Construction of ΦNM4γ4^*gfp*^

An overnight culture of *S. aureus* RN4220 harboring pAS23 (encoding the *gfp* gene with flanking sequences homologous to ΦNM4γ4 genome) was diluted 1:100 in fresh BHI supplemented with 5mM CaCl_2_ and appropriate antibiotics for selection. Cultures were grown shaking (220 RPM) at 37°C for 1.5 hrs and normalized by diluting to an OD_600_ of 0.1. A calculated volume of phage WT ΦNM4γ4 stock to give an MOI of 10 was added, and the culture was incubated overnight. The following day, the culture was pelleted, and the supernatant was collected and filtered through 0.45um *Supor*^®^ Membrane filters (VWR, Cat# 28143–352). To remove unedited phages, 100μl of a turbid overnight culture of *S. aureus* RN4220 harboring pAS22 (encoding a type II CRISPR-Cas system programmed to target the area of GFP insertion in the WT phage) was added to 10 ml of 50% BHI top agar supplemented with 5mM CaCl_2_ and plated onto solid BHI agar plates. 1:10 serial dilutions of filtered phage supernatant were then carried out and 2μl of each dilution was spotted onto the top agar in a phage titer assay. Plates were incubated at 37°C overnight.

10 phage plaques were selected the following day, resuspended in 30μl of BHI and 10μl of this was heated to 98°C for 10 minutes to extract the phage DNA. This was used as a template for PCR using the Phusion DNA Polymerase (Thermo Cat. #F530L) with oligos AS96 and AS98 to amplify across the GFP insertion area on the phage genome. PCR products were analyzed by agarose gel electrophoresis. The isolated phage that contained a band of the appropriate size for GFP insertion was titered once more on to top agar containing pAS22 (to ensure the complete elimination of any WT phage that may still be present). 10 phage plaques were selected the following day, resuspended in 30μl of BHI and 10μl of this was heated to 98°C for 10 mins to extract the phage DNA. PCR was once more carried out with AS96 and AS98, and the products analyzed with agarose gel electrophoresis. Isolated phage DNA containing the correct size band was then sent for Sanger sequencing and propagated in RN4220 top agar.

#### Spacer library construction and infection

For ΦNM4γ4 spacer library construction, a collection of 40,338 × 90-nt oligonucleotides were designed to contain a unique ΦNM4γ4-matching spacer, type III-A repeat homology, BsaI sites, and universal priming sites. For the ΦNM1γ6 spacer library construction, a similarly structured collection of 43,066 × 90-nt oligonucleotides was designed, each containing a unique ΦNM1γ6-matching spacer. The oligonucleotides were designed separately for the top and bottom strands of each phage to avoid unwanted homology during the downstream PCR step (see below) and purchased from Twist Biosciences. The libraries were made double-stranded by PCR with primers NA 484 and NA485, and the product was purified using MinElute PCR Purification Kit (Qiagen Cat. #28004). For each library, spacers were introduced into pNA15 via Golden Gate cloning with BsaI-HFv2 (New England Biolabs Cat. #R3733S) and T7 DNA ligase (New England Biolabs Cat. #M0318S), then electroporated into Endura Duo electrocompetent cells (Biosearch Technologies Cat. # 60242). Five hundred thousand colonies were pooled, and the plasmids were isolated and electroporated into *S. aureus* cells harboring either pNA1 (WT), pNA23 (*dcsm6*), pNA24 (*Cas10*^*HD*^), pNA28 (*dcsm6 dcsm3*) or pNA34 (*Cas10*^*HD*^ *dcsm3)*. Five hundred thousand CFU of each background were pooled and grown overnight. Overnight cultures were diluted 1:100 in 50 ml BHI supplemented with 5uM CaCl_2_ and appropriate antibiotics for selection. Cultures were grown shaking at 37°C until OD_600_ of 0.2 was reached. An uninfected sample (t_0_) was obtained by pelleting 10 ml of the culture and removing supernatant. Pellets were kept at −80°C until all time points were collected. MOI of 2 of ΦNM4γ4/ΦNM1γ6 phage was added to the sample before it was placed back shaking at 37°C. 5 hr post-infection 10 ml of the culture was pelleted and kept at −80°C. 24 hr post-infection 3 ml of the culture was pelleted and kept at −80°C.

#### Naïve spacer acquisition assay

Overnight cultures of *S. aureus* RN4220 containing pNA2 (WT), pNA3 (*dcsm6*) or pNA4 (*Cas10*^*HD*^) were diluted 1:100 in 10 mL of BHI and grown for 2.5 hr shaking at 37°C. OD600 was measured and used to calculate CFU per μL. For the infection, 1.3 billion CFUs were mixed with 2.6 PFUs of ΦNM4γ4^28^ for a starting MOI of 2. This mixture was added to 6 mL of melted 50% Heart Infusion Agar (HIA top agar) supplemented with 5 mM CaCl2. The top agar was poured onto a plate containing solidified BHI agar, and plates were then incubated at 37°C for 48 hr. To collect all colonies, top agar was scraped into a 50 ml falcon tube, supplemented with ~10 ml of liquid BHI, and incubated in a thermoblock set on 95°C, occasionally releasing vapor pressure until agar was solubilized. Cells were then pelleted and supernatant was removed quickly before resuming with spacer selection (described below).

#### Spacer selection and deep sequencing

Plasmids were isolated from *S. aureus* pellets with a modified protocol of QIAprep Spin Miniprep Kit (Qiagen Cat. #27104), as previously described^36^: Bacterial cell pellets were resuspended in 250 ul P1 buffer supplemented with lysostaphin (AMBI Products) at a final concentration of 107 ug/ml and incubated at 37°C for 30 min, followed by the standard QIAprep protocol. 250 ng of each sample were used as input for PCR, using the Phusion DNA Polymerase (Thermo Cat. #F530L) with primers NA162/NA163 for the spacer-library experiments, and with NA101/NA102 for naïve spacer acquisition (see Table S1 for a full list of primers used in this study).

Spacer library PCRs were analyzed by agarose gel electrophoresis, bands were excised and purified using QIAquick gel extraction kit (Qiagen Cat. #28704). Naïve spacer acquisition PCRs underwent cleanup with the MinElute PCR Purification Kit (Qiagen Cat. #28004) and size selection using PippnHT 3% cassette with a timed protocol set at extraction between 26 and 35 min. Size selected products were then prepared for sequencing with the TrueSeq Nano DNA Library Prep protocol (Illumina). For maintaining the small sized product, 2.2× Sample Purification Beads (Ilumina) were used after end repair. Illumina libraries underwent high-throughput sequencing with the MiSeq platform.

#### Phage RNA sequencing

Overnight culture of RN4220 was diluted 1:200 in 50 ml BHI supplemented with 5mM CaCl_2_ and grown 70 minutes shaking at 37°C. OD600 was measured and MOI of 2 of ΦNM4γ4 or ΦNM1γ6 was added to the culture before it was placed back shaking at 37°C. At 5, 15 and 30 minutes post-infection, 10 ml of the cultures were added to 50 ml falcon tubes containing 35 ml of ice-cold BHI and centrifuged at 4°C 120k rpm for 2 minutes. Supernatant was removed and pellets were resuspended in 100ul RNase free PBS supplemented with 100 μg/ml lysostaphin (AMBI Products), incubated for 5 min at 37°C, sarkosyl was added to a final concentration of 1%, gently flicking the tube to mix. RNA was purified from lysed pellets using the Zymo Direct-Zol RNA miniprep plus kit, and genomic DNA was removed by Ambion Turbo DNA-free kit. Illumina Ribo-Zero rRNA removal (Bacteria) kit was used to remove rRNA. Samples were prepared for sequencing using the TruSeq Stranded mRNA Library prep kit, beginning at the RNA fragmentation step. Illumina libraries underwent high-throughput sequencing with the MiSeq platform.

#### High-throughout sequencing data analysis

Spacers were extracted from MiSeq FASTQ files using a Python code (based on^28^) that finds all sequences flanked by two DR sequences. For spacer-library experiment, each spacer was counted by a python code and exported to excel. Spacer frequencies at each time-point were calculated and enrichment ratios were obtained by dividing each spacer frequency at 5 hr and 24 hr time by the frequency at t_0_.

For naïve spacer-acquisition assay, genome alignment maps were generated by alignment of all spacers to the ΦNM4γ4 genome using bowtie2. Genome positions covered by aligned spacers were counted and aggregated using the Python pysam package (version 0.15.3). Reads were analyzed at a single nucleotide resolution, and RPM values were calculated as phage reads per million total aligned reads. RNA-seq sequences underwent the same pipeline of analysis, and number of reads were normalized to account for the variability in total number of reads per time point.

#### Liquid culture growth assays

Overnight cultures started from three individual colonies of *S. aureus* RN4220 harboring the indicated pCRISPR were diluted 1:100 in BHI supplemented with 5uM CaCl_2_ and appropriate antibiotics for selection. Cultures were grown shaking at 37°C for 1.5 hr and re-diluted to OD_600_ of 0.1. 200ul of each culture was seeded into each well in a 96-well plate. A calculated volume of phage stock to give an MOI of either 1 or 10 (as indicated in the figure legend) was added to conditions with phage. No phage was added to control conditions. OD_600_ was measured every 10 minutes (TECAN Infinite 200 PRO) at 37°C with shaking.

#### Quantification of colony forming units

Overnight cultures started from three individual colonies of *S. aureus* RN4220 harboring the indicated pCRISPR were diluted 1:100 in BHI supplemented with 5uM CaCl_2_ and appropriate antibiotics for selection. Cultures were grown shaking at 37°C for 1.5 hr and re-diluted to OD_600_ of 0.1. Volumes were divided between no-phage and with-phage conditions. A 1 ml pre-infection aliquot was sampled from each culture. A calculated volume of phage stock to give MOI 10 was added to with-phage conditions. 1ml aliquots of all cultures were sampled at t_0_ and every 30 minutes for 4 hours. Each aliquot was spun down at 3700 rpm for 5 minutes. The supernatant was collected and stored for further phage titer calculation (see below). The pelleted cells were washed twice in 1ml BHI and finally resuspended in 100ul of BHI, serially diluted (1:10), and 5ul of each dilution was plated onto BHI agar plates supplemented with antibiotics for selection. Plates were incubated at 37°C overnight. Colonies were counted the following morning to calculate total CFU/ul of the culture.

#### Microscopy time course

Turbid overnight cultures of *S. aureus* RN4220 harboring the indicated pCRISPR were diluted 1:200 in BHI supplemented with 2.5mM CaCl_2_ and appropriate antibiotics for selection (5μl of culture into 1ml of media). Cells were loaded into microfluidic chambers in plates for bacterial use (Millipore Sigma, Cat #B04A-03) with the CellASIC ONIX2 microfluidic system. After cells became trapped in the chamber, they were supplied with BHI medium under a constant flow of 5 μl/hr for 2 hours to allow them to equilibrate. ΦNM4γ4^*gfp*^ stock at concentration of 10^7^ PFU/ul was added to the chamber for 20 min under a constant flow of 5 μl/hr. Flow was then switched back to BHI medium for the remainder of imaging (12–18 hours).

Phase contrast images were captured at x100 magnification every 2 min, using a Nikon Ti2e inverted microscope equipped with a Hamamatsu Orca-Fusion SCMOS camera with the temperature-controlled enclosure set to 37°C. GFP signal was imaged with a GFP filter set using an Excelitas Xylis LED Illuminator set to 5% power, with an exposure time of 200 ms. GFP images were captured at x100 magnification every 10 minutes. Timelapse images were aligned and processed using NIS Elements software v5.3. The timelapse files were converted to avi and mp4 videos using a 100ms frame rate. Timelapse labels were added to avi movies using the Label feature in Fiji v2.14.

#### Fluorescently activated single-cell sorting

Overnight cultures of *S. aureus* RN4220 harboring the indicated pCRISPR were diluted 1:100 in BHI supplemented with 5mM CaCl_2_ and appropriate antibiotics for selection. Cultures were grown shaking (220 RPM) at 37°C for 1 hr, normalized by diluting to an OD_600_ of 0.1, and infected with ΦNM4γ4^*gfp*^ phage at an MOI of 10. Cultures were grown shaking at 37°C for 1 hr. Cells were centrifuged at 3700 rpm for 5 min. The supernatant was discarded, and cells were washed twice in 5 ml of 1 x dPBS (Sigma-Aldrich, Cat #D8537). Cells were finally resuspended in 3ml of 1x dPBS.

This suspension of cells was loaded into the FACS Aria III flow cytometer (BD, USA) with a flow rate of 10,000 events per second. Bacterial cells were identified based on FSC, SSC, and fluorescence signal (GFP) using Diva software. Cells of interest were gated, and single cells were sorted into a 96-well plate containing 200μl of BHI supplemented with antibiotics. Plates were incubated overnight (TECAN Infinite 200 PRO) at 37°C with shaking (220 RPM). Further analysis was done using FlowJo v10.9.0. The initial GFP intensity of each single cell sorted was recorded using the index-sort module on the FACS Diva software and extracted with IndexSort v3.0.7 (BD FlowJo exchange). Proper biosafety measures and instrument maintenance were followed throughout the procedure to ensure accurate and contamination-free sorting.

#### Quantification of plaque forming units

Supernatants collected during the “quantification of colony forming units” assay (described above) were filtered with 0.45um *Supor*^®^ Membrane filters (VWR, Cat# 28143–352). 100ul of an overnight culture of *S. aureus* RN4220 (containing no CRISPR plasmid) was added to 10ml of BHI top agar supplemented with 5uM CaCl_2_ and plated on BHI agar plates. 1:10 serial dilutions of the filtered supernatants were carried out, and 2ul of each dilution was plated onto the top agar plates. Plates were incubated at 37°C overnight. Plaques were counted the following morning to calculate total PFU/ul of the cultures.

#### qPCR of phage DNA

For the time-course assay, infections were set up as described for the “quantification of colony forming units” (see above). A 15 ml pre-infection aliquot was sampled from each culture and a calculated volume of ΦNM4γ4^*gfp*^ to give MOI 10 was then added. 15 ml aliquots of all cultures were sampled at indicated timepoints up to 3 hours post-infection, after which 1 ml aliquots were sampled due to the increased cell density. Each aliquot was spun down at 10,000 rpm for 10 minutes. The pelleted cells were washed twice in BHI and stored at −80°C.

For the post-sorting assay, FACS was carried out as described above. Cells from regrown wells were collected and spun down at 10,000 rpm for 10 minutes. The pelleted cells were washed twice in BHI and stored at −80°C.

Bacterial and phage genomic DNA was extracted from pelleted cells following a modified Cold Spring Harbor Protocols^70^: pellets were thawed to room temperature and resuspended in 100ul of 50mM EDTA supplemented with 10ul of 1mg/ml lysostaphin, and incubated at 37°C for 20 minutes while shaking at 500 rpm. Then, the Wizard^®^ Genomic DNA Purification Kit (Promega, Cat# A1125) was used and the DNA pellet was resuspended in 50ul of Rehydration solution for 1 hour at 65°C. DNA concentration was determined using a nanodrop and gDNA was stored in −80°C.

Once gDNA was isolated, qPCR reactions were set up in triplicates using the Luna^®^ Universal qPCR Master Mix (NEB, Cat# M3003) and associated protocol. The fast cycling mode for standard quantification was selected on the QuantStudio 3 Real-Time PCR System (Applied Biosystems). To determine the amount of phage DNA in each sample, primers AS97 and AS99 were used to amplify a region of the GFP gene on the phage DNA. Ct values were normalized to the Ct values obtained with a host-specific primer set, AS518 and AS519, which amplified a small region on the bacterial chromosome to control for overall DNA content in each reaction. 1:10 serial dilutions of pCN57 were used to create the standard curve.

#### Characterization of escaper-phages

100μl of a turbid overnight culture of *S. aureus* cells containing the indicated pCRISPR with a given spacer sequence was added to 10ml of 50% BHI top agar supplemented with 5mM CaCl_2_ and the appropriate antibiotic for selection and plated onto solid BHI agar plates to provide a lawn on which to plaque escaper-phage. 1:10 serial dilutions were made of each filtered supernatant from the phage propagation assay described above (Total PFU quantification). 2μl of each dilution was spotted onto the top agar containing pCRISPR. Plates were incubated at 37°C overnight. Plaques were counted the following morning to calculate escaper PFU/ul.

To purify escaper-phage plaques, 10 escaper plaques were isolated from these plates and resuspended in 30μl BHI. 1:10 serial dilutions were carried out and 2μl of the resuspension was spotted once more on 50% BHI top agar containing pCRISPR to further select for the escaper-phages present. Plates were incubated at 37°C overnight.

Escaper-phage plaques were selected the following day, resuspended in 30μl of BHI and heated to 98°C for 10 mins to extract the phage DNA. This was used as a template for PCR using the Phusion DNA Polymerase (Thermo Cat. #F530L) with oligos AS393-AS398 to amplify across the spacer targets on the phage. PCR products were sent for Sanger sequencing to determine the sequence of escaper-phages.

#### Liquid growth assays with escaper-phages

Overnight cultures of *S. aureus* RN4220 harboring the indicated pCRISPR were diluted 1:100 in fresh BHI supplemented with 5mM CaCl_2_ and appropriate antibiotics for selection. Cultures were grown shaking (220 RPM) at 37°C for 1.5 hrs and normalized by diluting to an OD_600_ of 0.2. A calculated volume of phage stock to give an MOI of 10 was added, and cultures were incubated for another 1 hr at 37°C shaking. Cultures were then re-diluted to OD_600_ of 0.1 to resume the exponential growth phase post-infection. 200μl of each culture was seeded into a 96-well plate (Cellstar). OD600 was measured every 10 minutes (TECAN Infinite 200 PRO) at 37°C with shaking (220 RPM) for 24 hrs.

#### Sequential phage infections

Overnight cultures started from three individual colonies of *S. aureus* RN4220 harboring the indicated pCRISPR were diluted 1:100 in fresh BHI supplemented with 5mM CaCl_2_ and appropriate antibiotics for selection. Cultures were grown shaking (220 RPM) at 37°C for 1.5 hrs and normalized by diluting to an OD_600_ of 0.2. The calculated volume of ΦNM4γ4 phage to give an MOI of 10 was then added to conditions that required prior activation with a targeted phage. Cultures were then incubated shaking at 37°C for 1 hour and were normalized once more by diluting to an OD_600_ of 0.1.

At this point, 200μl of each culture was seeded into a 96-well plate (Cellstar). A calculated volume of non-targeted Φ12γ3 or Φ80α phage stock was then added to all the wells to give an MOI of 10 or 1 respectively. OD_600_ was measured every 10 minutes (TECAN Infinite 200 PRO) at 37°C with shaking (220 RPM) for 24 hrs, and these measurements were used to generate growth curves.

### QUANTIFICATION AND STATISTICAL ANALYSIS

Statistical tests and generation of graphs was done in GraphPad Prism 10.2.0. Description of statistical tests, value of *n*, and dispersion and precision measures used can be found in the figure legends. Significance was defined as p<0.05. Graph formatting was done using Adobe Illustrator 28.2.

## Supplementary Material

1 **Document S1**. Figures S1 – S6

2 **Supplementary Table S1. Strains, plasmids, oligonucleotides, cloning strategies, and NGS data used in this paper**. Related to STAR Methods and Figures 1–4 and S1–S4. (Tab A) Bacterial strains and bacteriophages used in this study. (Tab B) Plasmids used in this study. (Tab C) Oligonucleotide sequences used in this study. (Tab D) Cloning strategies for plasmids that were constructed in this study. (Tab E) Annotated reads from NGS of ΦNM4γ4 spacer library assays, used in panels 1C, 2A, 3A, S1A-B, S1D, S2A-D, S3A-B. F) Frequencies of RNA-seq reads of ΦNM4γ4, used in panels S1C-D. (Tab G) Frequencies of RNA-seq reads of ΦNM1γ6, used in panel S4B. (Tab H) Annotated reads from NGS of ΦNM1γ6 spacer library assays, used in panels S4C-L. (Tab I) Annotated reads from NGS of ΦNM4γ4 WT spacer acquisition assays, used in panel 4A. (Tab J) Annotated reads from NGS of ΦNM4γ4 d*csm6* spacer acquisition assays, used in panel 4C. (Tab K) Annotated reads from NGS of ΦNM4γ4 *cas10*^HD^ spacer acquisition assays, used in panel 4B.

3 Video S1. Fluorescence microscopy of RN4220 cells harboring pCRISPR and *spc15* infected with ΦNM4γ4^*gfp*^, related to Fig 1 and Fig S1.

4 Video S2. Fluorescence microscopy of RN4220 cells harboring pCRISPR and *spc36* infected with ΦNM4γ4^*gfp*^, related to Fig 1 and Fig S1.

5 Video S3. Fluorescence microscopy of RN4220 cells harboring pCRISPR and *spc47* infected with ΦNM4γ4^*gfp*^, related to Fig 1 and Fig S1.

6 Video S4. Fluorescence microscopy of RN4220 cells harboring pCRISPR(d*csm6*) and *spc15* infected with ΦNM4γ4^*gfp*^, related to Fig 2 and Fig S2.

7 Video S5. Fluorescence microscopy of RN4220 cells harboring pCRISPR(d*csm6*) and *spc36* infected with ΦNM4γ4^*gfp*^, related to Fig 2 and Fig S2.

8 Video S6. Fluorescence microscopy of RN4220 cells harboring pCRISPR(d*csm6*) and *spc47* infected with ΦNM4γ4^*gfp*^, related to Fig 2 and Fig S2.

9 Video S7. Fluorescence microscopy of RN4220 cells harboring pCRISPR(*cas10*^HD^) and *spc15* infected with ΦNM4γ4^*gfp*^, related to Fig 3 and Fig S3.

10 Video S8. Fluorescence microscopy of RN4220 cells harboring pCRISPR(*cas10*^HD^) and *spc36* infected with ΦNM4γ4^*gfp*^, related to Fig 3 and Fig S3.

11 Video S9. Fluorescence microscopy of RN4220 cells harboring pCRISPR(*cas10*^HD^) and *spc47* infected with ΦNM4γ4^*gfp*^, related to Fig 3 and Fig S3.

Supplementary File 1

## Figures and Tables

**Table 1.** Spacers found in staphylococcal type III-A CRISPR-Cas systems that match annotated staphylococcal phages.

Spacer sequence^#^	Phage	Target gene	Exp.*
GAGAACTTAATTGCATTATCAAATGTATATGCTGGATTCCA	phi66	SPV66_ORF017	late
GAGAACCCGAATTTTGATTCTTTGTTTGTAAATAATGCTC	SAP-2	SAP2_gp03	early
GAGAACCACGCTGTAGTGAAGTATAGAAACGGCATGAGTACAA	52A	ST52AORF079	late
AGTCAATATAAAGACAATACTTTTTACGCTTATATT	phiIBB-SEP1	FDH45_gp044	NA
AATGAAATTTATCAAAACTATAGAAAACTTATTAG	vB_Sau_CG	vBSauCG_155	late
TGGTTTAAGTTTGTCATTATAATCAATCCTTTTTCTT	pSa-3	pSa3_007	early
GTTTTTCATAGTTAATCAATCCCTTTTCTTTTTT	pSa-3	pSa3_067	NA
TTAAATCTTTGATTGCTCTTAGCTCTAGTTATGTAT	Bacterio88	ST88ORF056	late
CACGCTGTAGTGAAGTATAGAAACGGCATGAGTACAAT	52A	ST52AORF079	late
CTTCCGAATCCATTTCAGCGCAATAAACA	SpT99F3	SpT99F3_045	NA
ACTCACTTGTAAATTCCTCCACTTGCTCTA	Bacterio2638A	ST2638AORF019	late
CTGGAATAACCACAAAGCCAGAGTCAGTTT	phi575	NA	NA
ACGTTAGATTTGCAGGTGTTAAGCACGGCT	vB_SepS_SEP9	SEP9_018	NA
TAGAATGTTATTATCTAAGTGGTCGATGTATTCC	BacterioG1	ORF135-ORF092	late
TTTTCTTTAACTGTTTTTACTGCCCATTTAATAGT	phiIPLA-C1C	AVU40_gp150	late
AAGTTAACGGCATTACCTAATAAAAATATTTTAGG	SAP-2	SAP2_gp08	early
GTTTTTCATAGTTAATCAATCCCTTTTCTTTTTT	vB_SauM_Remus	O151_gp047	NA
TATGTATTGATCTCGATTCTCGTTAGTTTCTAAATT	phiNM1	SAPPV1_gp32	early
CACGCTGTAGTGAAGTATAGAAACGGCATGAGTACAAT	SP6	SP6_0011	early
TAGTAATAATTGTCTCATTTGCATACGTTACATCGAT	CNPH82	cn20	NA
TAGAATGTTATTATCTAAGTGGTCGATGTATTCC	S25–3	X600_gp192	early
AAGTTAACGGCATTACCTAATAAAAATATTTTAGG	S13’	ORF9	early
CCAAACCATTTAGCACGATATTTATTAAAACCATA	K	gp011	early
TATTTTTCTCCTTTAGCAATCATTCTGTCTAGTAC	S25–3	X600_gp187	early

# Spacer sequences were obtained from previous bioinformatic analyses^17,18^.

* Time of expression was inferred from the position on the target gene in the viral genome. NA: not assigned (difficult to determine time of expression).

**Table:** KEY RESOURCES TABLE

REAGENT or RESOURCE	SOURCE	IDENTIFIER
**Bacterial and virus strains**		
S. *aureus* RN4220	Kreiswirth et al.^29^	N/A
Endura Duo electrocompetent cells	Biosearch Technologies	Cat#60242
ΦNM4γ4	Goldberg et al.^26^	N/A
ΦNM4γ4^*gfp*^	This study	N/A
ΦNM1γ6	Goldberg et al.^26^	N/A
Φ12γ3	Modell et al.^43^	N/A
Φ80α*vir*	Banh, Roberts et al.^35^	N/A
		
**Chemicals, peptides, and recombinant proteins**		
Chloramphenicol	G-biosciences	Cat#RC-167
Spectinomycin dihydrochloride pentahydrate	Goldbio	Cat#S-140–5
BsaI-HF^®^v2	New England Biolabs	Cat#R3733S
T7 DNA ligase	New England Biolabs	Cat#M0318S
Lysostaphin	AMBI Products	Cat#LSPN-50
Dulbecco’s Phosphate Buffered Saline	Millipore Sigma	Cat#D8537
		
**Critical commercial assays**		
MinElute PCR Purification kit	Qiagen	Cat#28004
QIAprep Spin Miniprep kit	Qiagen	Cat#27104
QIAquick gel extraction kit	Qiagen	Cat#28704
Zymo Direct-Zol RNA miniprep plus kit	fisherscientific	Cat#50-444-628
TURBO DNA-*free*^™^ kit	Thermofisher	Cat#AM1907
Illumina Ribo-Zero Plus rRNA Depletion kit	Illumina	Cat#20036696
TruSeq Stranded mRNA Library prep kit	Illumina	Cat#20020595
TruSeq Nano DNA High Throughput Library Prep kit	Illumina	Cat#20015965
2 % Agarose Gel Cassette, Pippin HT	Sage Science	Cat#HTC2010
Wizard^®^ Genomic DNA Purification kit	Promega	Cat#A1125
Luna^®^ Universal qPCR Master Mix	New England Biolabs	Cat#M3003
Phusion^™^ High-Fidelity DNA Polymerase kit	Thermofisher	Cat#F530L
NEBuilder^®^ HiFi DNA Assembly Master Mix	New England Biolabs	Cat#2621L
CellASIC ONIX plate for bacteria cells	Millipore Sigma	Cat#B04A-03
		
**Deposited data**		
ΦNM4γ4 spacers deep sequencing and RNA-seq data	This paper	PRJNA1075789
ΦNM1γ6 spacers deep sequencing and RNA-seq data	This paper	PRJNA1173170
		
**Oligonucleotides**		
See Supplementary Tables S1 for description of all oligos used in this study		
		
**Recombinant DNA**		
See Supplementary Table S1 for cloning strategies used to generate plasmids		
pNP54	Pyenson et al.^32^	N/A
pCN57	Charpentier et al.	doi: 10.1128/AEM.70.10.6076-6085.2004
pAS04	This study	N/A
pAS20	This study	N/A
pAS21	This study	N/A
pAS22	This study	N/A
pAS23	This study	N/A
pAS53	This study	N/A
pAS54	This study	N/A
pAS55	This study	N/A
pAS56	This study	N/A
pAS61	This study	N/A
pAS66	This study	N/A
pAS67	This study	N/A
pAS69	This study	N/A
pAS70	This study	N/A
pAS71	This study	N/A
pAS72	This study	N/A
pAS73	This study	N/A
pAS74	This study	N/A
pAS78	This study	N/A
pLM554	Hatoum-Aslan et al.^9^	N/A
pLZ12spec	Husmann et al.	doi: 10.1128/iai.63.1.345-348.1995
pGG79-F	Goldberg et al.^26^	N/A
pDB114	Bikard et al.	doi: 10.1038/nbt.3043
pC194	Horinouchi et al.^44^	N/A
pGG-Bsal-R	Samai et al.^13^	N/A
pFNMB0-mCherry-2	Brodmann et al.	doi: 10.3389/fcimb.2018.00284
pNA1	Aviram et al.^36^	N/A
pNA2	Aviram et al.^36^	N/A
pNA3	This study	N/A
pNA4	This study	N/A
pNA15	This study	N/A
pNA23	This study	N/A
pNA24	This study	N/A
pNL03	This study	N/A
pNL09	This study	N/A
pNL223	This study	N/A
pNL224	This study	N/A
pNL225	This study	N/A
		
**Software and algorithms**		
Prism 10.2.0	GraphPad	https://www.graphpad.com/features
Bowtie2	Bioconda	https://anaconda.org/bioconda/bowtie2
Python	Python	https://www.python.org/
Python scripts for extracting spacer sequences from NGS data	Based on Heler et al. 2005	https://doi.org/10.1038/nature14245
BD FACSDiva^™^ Software	BD Biosciences	https://www.bdbiosciences.com/en-us/products/software/instrument-software/bd-facsdiva-software
FlowJo v10.9.0	BD Biosciences	https://www.flowio.com/solutions/flowio
Index sort v3.0.7	BD Biosciences, FlowJo Exchange	https://www.flowio.com/exchange/#/plugin/profile?id=20
NIS elements	Nikon	https://www.microscope.healthcare.nikon.com/products/software/nis-elements
Fiji v2.14	ImageJ	https://imagei.net/software/fiii/
Illustrator v28.7.1	Adobe	https://www.adobe.com/products/illustrator/
		
**Other**		
Infinite^®^ 200 PRO	TECAN	N/A
CellASIC ONIX2	Millipore Sigma	Cat#CAX2-S0000
Eclipse Ti2-E Inverted Microscope	Nikon	N/A
BD FACSAria^™^ III Cell Sorter	BD, USA	N/A
PippinHT	Sage Science	N/A
MiSeq System	Illumina	N/A

## Highlights

- CRISPR-Cas10 immunity induces growth arrest of the infected cell
- Cas10 ssDNase activity is required for growth arrest relief and regrowth after infection
- Growth arrest provides broad-spectrum immunity and population level protection
