# Supplementary material for: Cas10 relieves host growth arrest to facilitate spacer retention during type III-A CRISPR-Cas immunity
Source: Cell Host Microbe. Author manuscript; Available in PMC 2025 Apr 11. (PMC11708336; doi:10.1016/j.chom.2024.11.005)
Supplement: 1 — Document S1. Figures S1 – S6 [file NIHMS2035246-supplement-1.pdf]

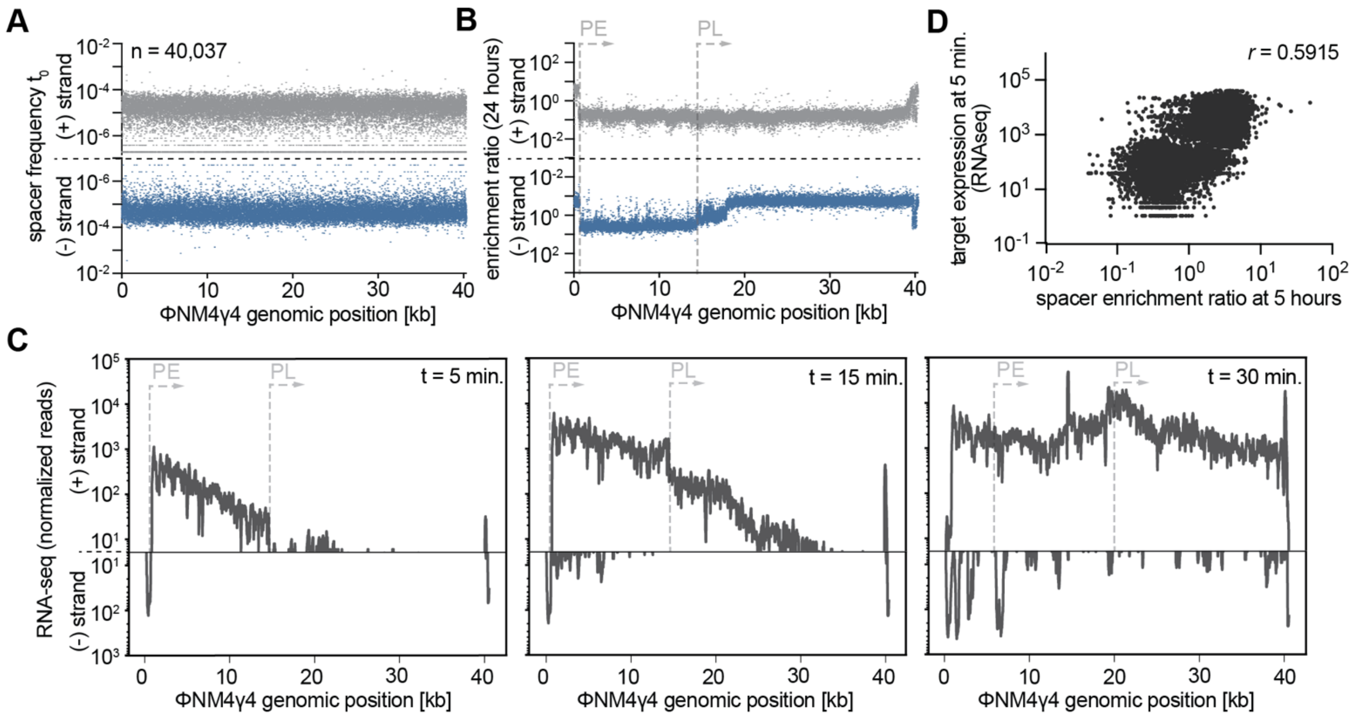

E

spacer	sequence (5’-3’)
spc15	GGAATTTGTATTCTTCTTGAAGTTTTCCTATTAAT
spc17	CGTTATCTTTCTGGTATCTTGTAATGTATTGCATT
spc19	GGTATAATTTTATTATCGCTATTGCGTTAGATTGG
spc29	TGCTTTTTTAATTTATAATTACATTTAGACGCTAA
spc36	CGTTAAATAGTCATCATCATTTCTAAAATTGTCCA
spc47	ACCACTAGTTGAAGTGTTGTAAGGTGATTTAGTAC

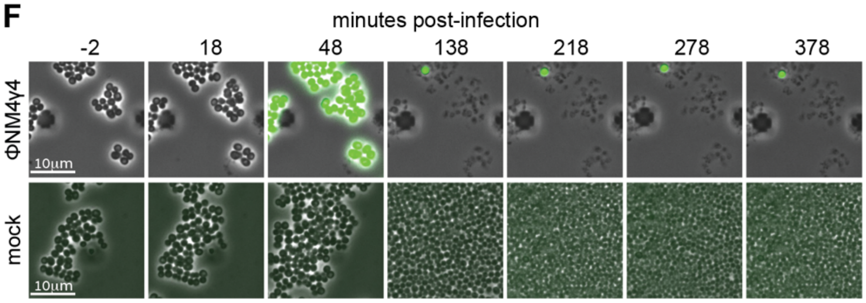

Figure S1. Targeting of the ΦNM4γ4 phage by the *S. epidermidis* type III-A CRISPR-Cas system. Related to Figure 1. **(A)** Spacer frequency, calculated as the fraction of spacer reads, for the library of spaces introduced into wild-type pCRISPR. Spacer sequences matching the plus and minus strands of the ΦNM4γ4 genome are plotted separately. **(B)** Enrichment ratio of spacers targeting the plus or minus strands of the ΦNM4γ4 DNA 24 hours after phage infection of staphylococci carrying pCRISPR, plotted according to their genomic position. **(C)** Abundance (in normalized reads) of ΦNM4γ4 RNA-seq reads, obtained at 5, 15 or 30 minutes after infection, mapped to the viral genome. **(D)** Correlation of target transcript expression (5 minutes after infection; normalized RNA-seq reads) and enrichment of the corresponding targeting spacer (5 hours after infection). Pearson *r* coefficient is shown. **(E)** Spacer sequences used in this study. **(F)** Time-course fluorescence microscopy of staphylococci at the indicated times after infection with ΦNM4γ4^{GFP} or a mock infection (media only).

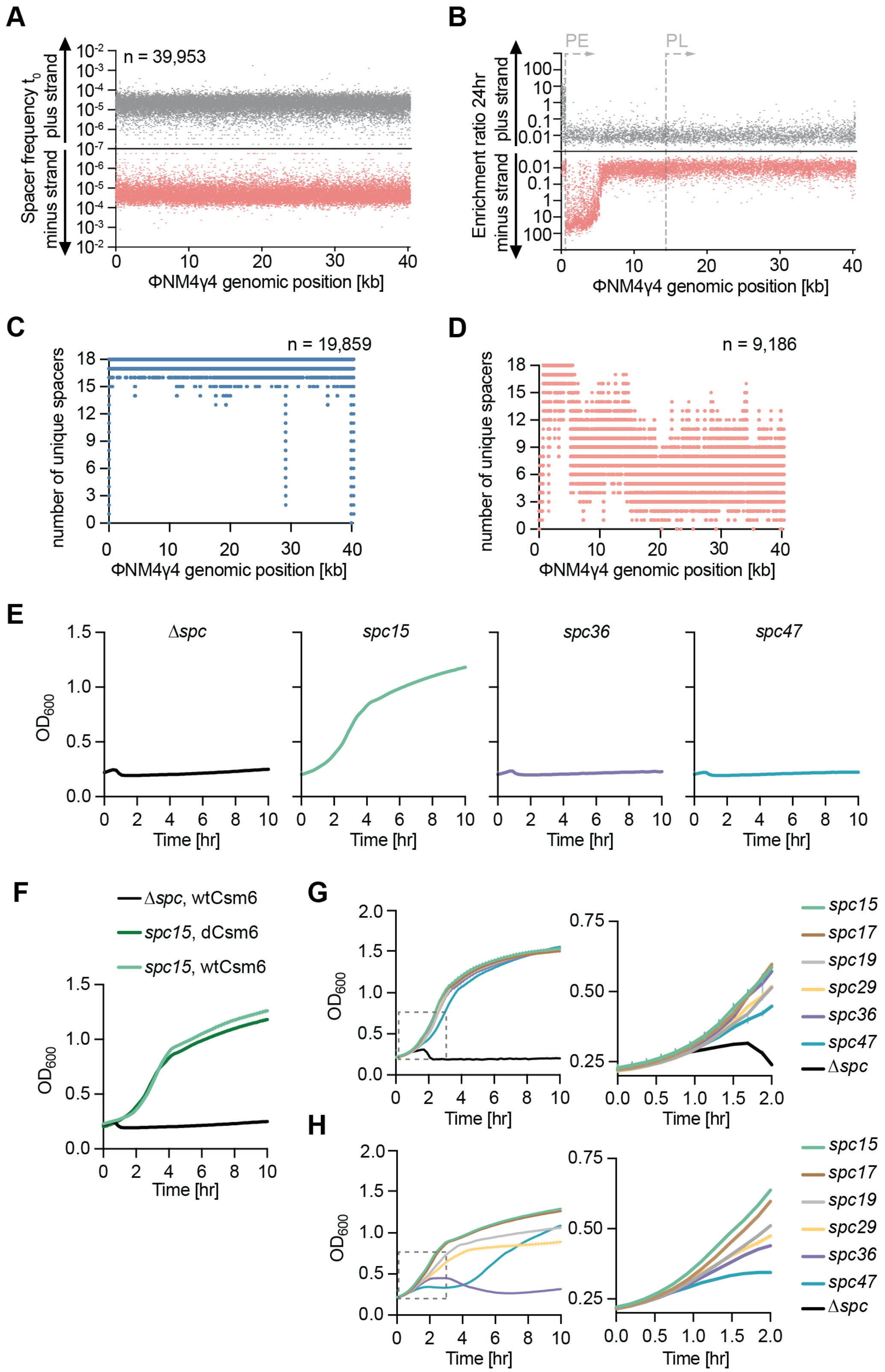

Figure S2. Targeting of the ΦNM4γ4 phage by a *S. epidermidis* type III-A CRISPR-Cas system carrying the d*csm6* allele. Related to Figure 2. **(A)** Spacer frequency, calculated as the fraction of spacer reads, for the library of spacers cloned into pCRISPR(d*csm6*). Spacer sequences matching the plus and minus strands of the ΦNM4γ4 genome are

plotted separately. **(B)** Enrichment ratio of spacers targeting the plus or minus strands of the ΦNM4γ4 DNA 24 hours after phage infection of staphylococci carrying pCRISPR(d*csm6*), plotted according to their genomic position. **(C)** Number of unique spacers detected after NGS of the pCRISPR library infected with ΦNM4γ4 for 24 hours, plotted across the viral genome. **(D)** Same as **(C)** for pCRISPR(d*csm6*). **(E)** Individual growth curves shown in Figure 1D. **(F)** Mean (± SD, n = 3 biological replicates) OD_{600} values of staphylococcal cultures harboring a pCRISPR or a pCRISPR(d*csm6*) plasmid programmed with *spc15,* after infection with ΦNM4γ4 at MOI 10. Infection of control strain harboring a CRISPR system lacking a targeting spacer is shown. **(G)** Mean (± SD, n = 3 biological replicates) OD_{600} values of staphylococcal cultures harboring a pCRISPR programmed with spacers 15, 17, 19, 26, 36 and 47; or lacking a targeting spacer (Δ*spc*), at different times after infection with ΦNM4γ4 at an MOI of 1. The area within the dashed line is shown in the right graph. **(H)** Same as **(G)** for cultures harboring pCRISPR(d*csm6*).

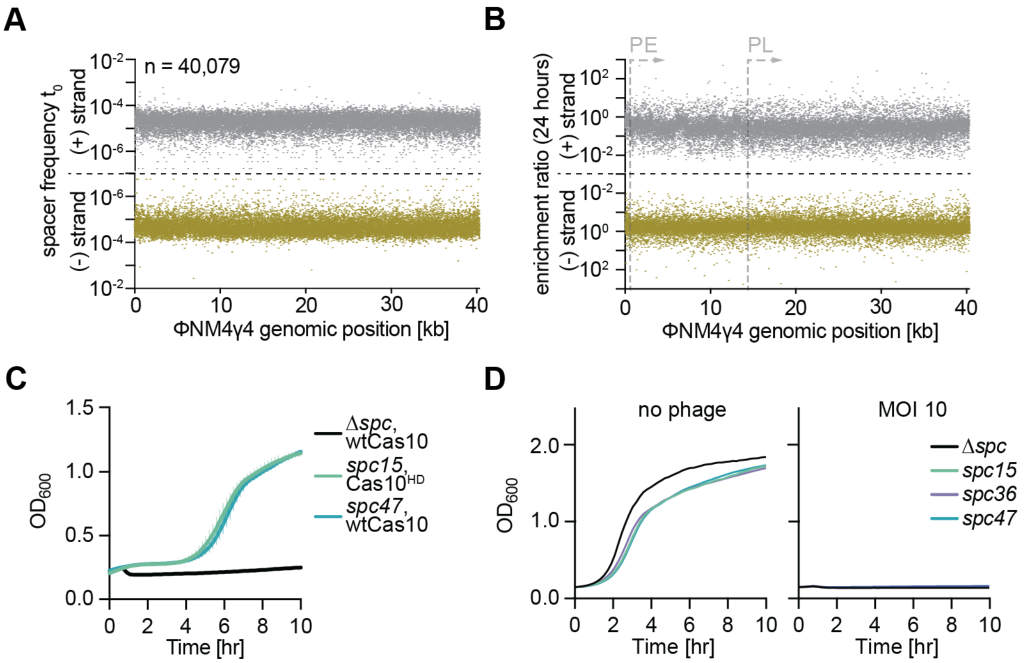

Figure S3. Targeting of the ΦNM4γ4 phage by a *S. epidermidis* type III-A CRISPR-Cas system carrying the *cas10*^{HD} allele. Related to Figure 3**. (A)** Spacer frequency, calculated as the fraction of spacer reads, for the library of spacers cloned into pCRISPR(*cas10*^{HD}). Spacer sequences matching the plus and minus strands of the ΦNM4γ4 genome are plotted separately. **(B)** Enrichment ratio of spacers targeting the plus or minus strands of the ΦNM4γ4 DNA 24 hours after phage infection of staphylococci carrying pCRISPR(*cas10*^{HD}), plotted according to their genomic position. **(C)** Mean (± SD, n = 3 biological replicates) OD_{600} values of staphylococcal cultures harboring a pCRISPR or a pCRISPR(*cas10*^{HD}) plasmid programmed with *spc47* or *spc15*, respectively, after infection with ΦNM4γ4 at MOI 10. Infection of control strain lacking a targeting spacer (Δ*spc*) is shown. **(D)** Mean (± SD, n = 3 biological replicates) OD_{600} values of staphylococcal cultures harboring a pCRISPR(*cas10*^{HD}, d*csm6*) plasmid programmed with the indicated spacers (*spc15, spc36, spc47,* Δ*spc*) either uninfected (left) or after infection with ΦNM4γ4 at MOI 10 (right).

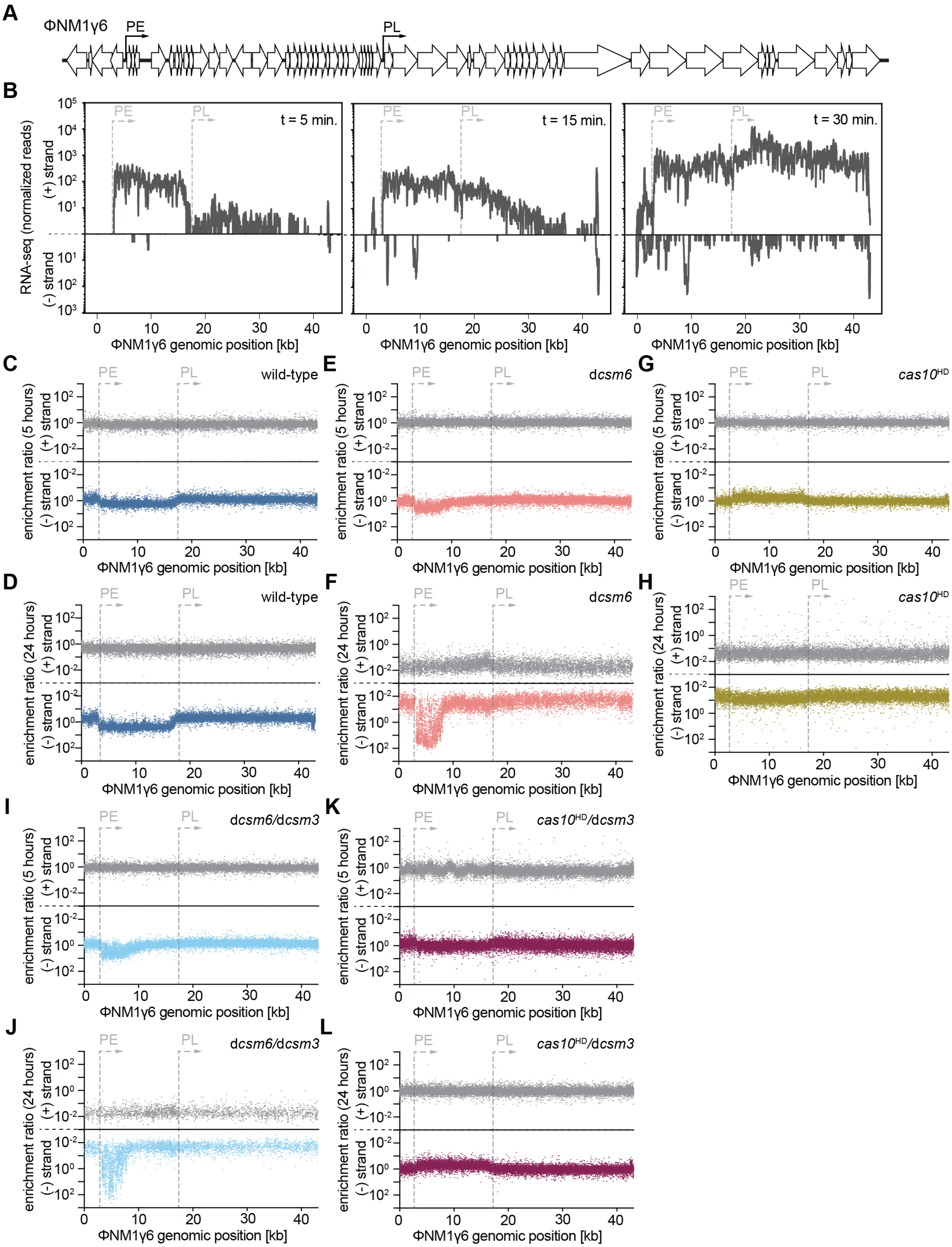

A
ΦNM1γ6
PE
PL
B
t = 5 min.
t = 15 min.
t = 30 min.
RNA-seq (normalized reads)
(+) strand
(-) strand
ΦNM1γ6 genomic position [kb]
C
wild-type
D
wild-type
E
dcsm6
F
dcsm6
G
cas10HD
H
cas10HD
I
dcsm6/dcsm3
J
dcsm6/dcsm3
K
cas10HD/dcsm3
L
cas10HD/dcsm3
enrichment ratio (5 hours)
enrichment ratio (24 hours)

Figure S4. Targeting of the ΦNM1γ6 phage by the *S. epidermidis* type III-A CRISPR-Cas system. Related to Figure 4. **(A)** Schematic representation of the ΦNM1γ6 phage genome. Arrows indicate the two promoters, PE and PL, that drive transcription of early- and late-expressed genes. **(B)** Abundance (in normalized reads) of ΦNM1γ6 RNA-seq reads, obtained at 5, 15 or 30 minutes after infection, mapped to the phage genome. **(C, D)** Enrichment ratio of spacers targeting the plus or minus strands of the ΦNM1γ6 DNA, 5 hours **(C)** or 24 hours **(D)** after phage infection of staphylococci carrying pCRISPR, plotted according to their genomic position. **(E, F)** Same as **(C, D)** for staphylococci carrying pCRISPR(d*csm6*). **(G, H)** Same as **(C, D)** for staphylococci carrying pCRISPR(*cas10*^{HD}). **(I, J)** Same as **(C, D)** for staphylococci carrying pCRISPR(d*csm6*, d*csm3*). **(K, L)** Same as **(C, D)** for staphylococci carrying pCRISPR(*cas10*^{HD}, d*csm3*).

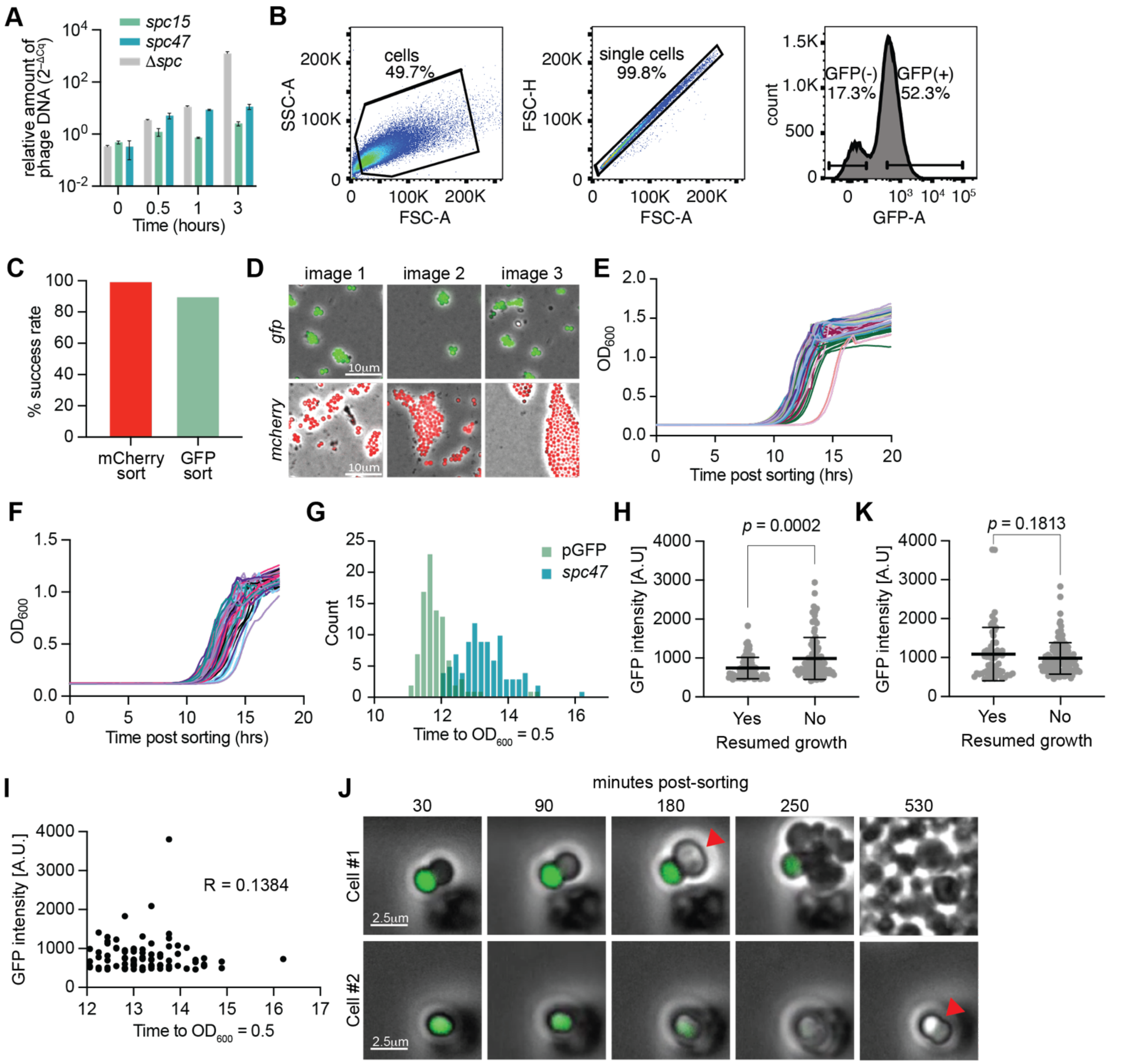

Figure S5. Evaluation of the exit from growth arrest of infected cells expressing GFP using single-cell analyses. Related to Figure 5. **(A)** Mean (± SD, n = 3 technical replicates) relative abundance of phage genome, as measured by qPCR, sampled at the indicated timepoints after infection of staphylococci harboring pCRISPR programmed with the indicated spacers (*spc15, spc47, ∆spc*) with ΦNM4γ4^{gfp} at MOI 10. **(B)** Gates used for sorting cells from debris (left), single cells from doublets (center) and GFP positive cells (right). **(C-D)** Single-cell sorting control. A mixture of cells expressing mCherry or GFP from a plasmid were sorted into each well of a 96 well plate using gating strategies for either mCherry or GFP. A successful sort was classified as a well that only contained the fluorescent protein that was sorted for, which was determined by examining the cells under the microscope. **(C)** Percentage of successful sorts compared to the total number of wells sorted for each is plotted. **(D)** Images of GFP- and mCherry-sorted cells. Three representative images are shown for each condition. **(E)** Growth of sorted single cells harboring the GFP-expressing plasmid control within a 96-well plate measured as OD_{600} values over time after sorting. **(F)** Growth of sorted single cells (harboring pCRISPR programmed with *spc47* and infected with ΦNM4γ4^{gfp}) within a 96-well plate measured as OD_{600} values over time after sorting. **(G)** Number of wells that with OD_{600} 0.5 values after sorting and regrowth, distributed across the time

required to reach that value, for the growth curves shown in **(E)** and **(F)**. **(H)** Mean (± SD, n=2 independent experiments of 96-well sorted cells) initial GFP intensity of ΦNM4γ4^{gfp}-infected staphylococci harboring pCRISPR programmed with *spc47* during indexed cell sorting, categorized based on growth outcome (*p* value obtained by unpaired t-test). **(G)** Growth of sorted single cells harboring pCRISPR programmed with *spc47* and infected with ΦNM4γ4^{gfp} within a 96-well plate, measured as OD_{600} values over time after sorting. **(I)** Correlation of initial GFP intensity and the time (hours) a given well reached OD_{600} of 0.5. Pearson *R* coefficient is depicted on the plot. **(J)** Time-course fluorescence microscopy of staphylococci programed with *spc47* after infection with ΦNM4γ4^{gfp} at the indicated times after GFP-sorting. **(K)** Same as **(H)** but using staphylococci harboring pCRISPR(*cas10*^{HD}) programmed with *spc47*.

A

Spacer	Target location (*)	Target deletion (*)	Deletion size (bp)	# of escapers
spc15	4,236...4,270	3,807...5,055	1,249	2/10
spc15	4,236...4,270	4,544...6,308	1,765	7/10
spc15	4,236...4,270	4,544...6,430	1,887	1/10
spc36	12,909...12,943	12,930...12,932	3	4/10
spc36	12,909...12,943	12,926...13,015	90	1/10
spc36	12,909...12,943	12,931...13,023	93	2/10
spc36	12,909...12,943	12,894...12,943	50	2/10
spc36	12,909...12,943	12,807...13,126	320	1/10

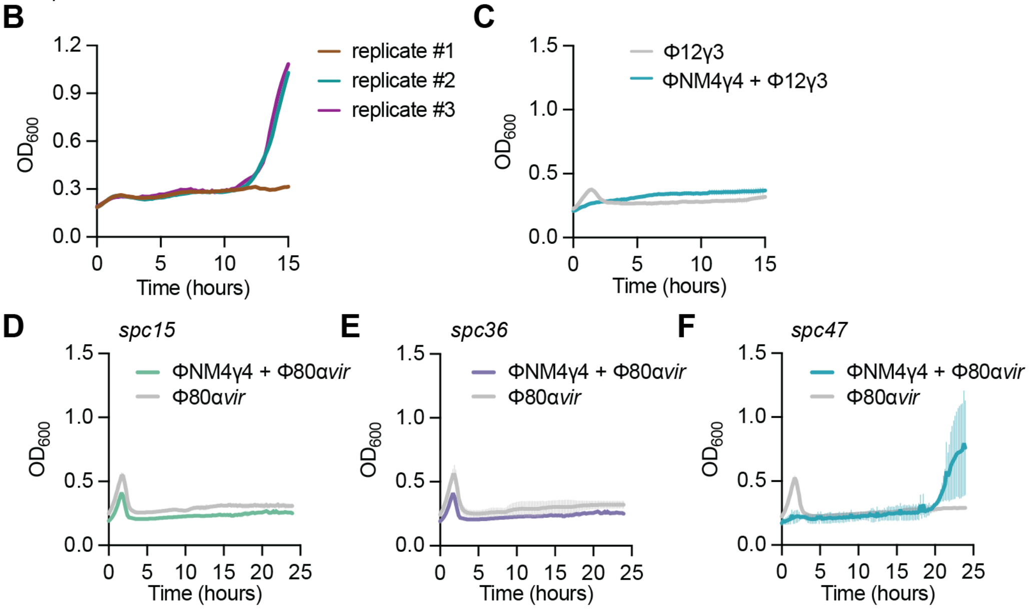

Figure S6. Type III-A immunity protects staphylococci against mixed phage infections. Related to Figure 6. **(A)** The location of target deletions from sequenced phage escapers shown in Figure 6A in relation to spacer target sites. (*) numbers indicate the genomic coordinates of ΦNM4γ4 (GenBank accession KP209285.1). **(B)** Growth of individual culture replicates shown in Figure 6F. **(C)** Mean (± SD, n = 3 biological replicates) OD_{600} values of staphylococcal cultures harboring pCRISPR(d*csm6*) programmed with *spc47*, infected with ΦNM4γ4 at MOI 10 for one hour and then infected with Φ12γ3 at MOI 10. Infection with Φ12γ3 without prior treatment is shown as control. **(D)** Mean (± SD, n = 3 biological replicates) OD_{600} values of staphylococcal cultures harboring pCRISPR programmed with *spc15*, infected with ΦNM4γ4 at MOI 10 for one hour and then infected with Φ80α*vir* at MOI 1. Infection with Φ80α*vir* without prior treatment is shown as control. **(E)** Same as **(D)** but following infection of cultures programmed with *spc36*. **(F)** Same as **(D)** but following infection of cultures programmed with *spc47*.
